# Bootstrap-enhanced regularization addressing multicollinearity and skewness in high-dimensional immunophenotyping data

**DOI:** 10.1186/s12859-026-06586-x

**Published:** 2026-08-06

**Authors:** Xiaoru Dong, Apoorva Goyal, Muxuan Liang, Maigan A. Brusko, Todd M. Brusko, Rhonda Bacher

**Affiliations:** 1 Department of Public Health Sciences, College of Dentistry, Texas A&M University, Dallas, TX 75246 USA; 2 Department of Biomedical Sciences, College of Dentistry, Texas A&M University, Dallas, TX 75246 USA; 3 Department of Biostatistics, College of Public Health and Health Professions, University of Florida, Gainesville, FL 32610 USA; 4 Diabetes Institute, University of Florida, Gainesville, FL 32610 USA; 5 Department of Biostatistics, University of Texas MD Anderson Cancer Center, Houston, TX 77030 USA; 6 Department of Pathology, Immunology, and Laboratory Medicine, College of Medicine, University of Florida, Gainesville, FL 32610 USA

**Keywords:** Multicollinearity, Bootstrapping, Regularized regression, Immunophenotyping data

## Abstract

**Background:**

Accurate identification and estimation of variables associated with outcomes or disease states are critical for advancing diagnosis, prognosis, and precision medicine in biomedical research. Regularized regression techniques, such as lasso, are widely employed to enhance interpretability by reducing model complexity and identifying significant variables. However, these methods face two major challenges: (1) the exclusion of important variables due to high correlation with included predictors, and (2) the presence of skewness in human biomedical datasets, which violates key statistical assumptions. Current approaches that fail to address these issues simultaneously may lead to biased interpretations and unreliable coefficient estimates. To overcome these limitations, we propose an enhanced two-step approach, the Bootstrap-Enhanced Regularization Method (BERM).

**Results:**

BERM outperformed existing regularization methods in variable selection, achieving the highest overall balanced accuracy while maintaining competitive coefficient estimation performance across a range of simulated sparsity, noise, and dimensionality scenarios. We further demonstrated the effectiveness of BERM by applying it to a human immunophenotyping dataset to identify important immune parameters in the autoimmune disease, type 1 diabetes.

**Conclusion:**

BERM is a robust approach for variable selection and coefficient estimation in complex biomedical datasets. Its consistent performance across a wide range of data conditions supports more reliable identification of important variables. An open-source implementation of BERM is available as an R package on GitHub (https://github.com/xiaorudong/berm).

**Supplementary Information:**

The online version contains supplementary material available at https://doi.org/10.1186/s12859-026-06586-x.

## Background

With advances in high-throughput technologies in biomedical research, the potential for precision medicine and identifying predictive biomarkers is more achievable than ever before. Techniques such as mass cytometry, high-dimensional flow cytometry, and CITE-seq enable the characterization of cellular subsets and activation states based on 20 to 50 individual surface and intracellular markers measured simultaneously [6, 24, 28]. The combination or interaction of these parameters, representing hundreds to thousands of cellular variables, are of particular interest in identifying disease-associated cellular signatures. Immune cells, for instance, are broadly defined in peripheral blood by canonical cell surface markers, including CD3 (T cells), CD19 (B cells), CD11c (dendritic cells [DCs]), CD56 (natural killer [NK] cells), CD68 (macrophages). Combinations of these and other markers further distinguish different cell subsets and functional statuses [7], enabling the identification of alterations in cell compositions associated with vaccination, infection, autoimmunity, and cancer [3, 8, 11, 18]. As the number of parameters increases with technological advances in instrumentation and reagents, immunophenotyping datasets have become increasingly high-dimensional, with the number of measured variables larger relative to the number of samples. Consequently, the analysis of immunophenotyping data increasingly requires advanced statistical methods. For instance, the high dimensionality leads to challenges in identifying and quantifying which systematic variations in cell states are associated with time, lifespan, and disease [20]. Existing analysis methods are largely geared toward classification and emphasize dimension reduction to compress the complex feature-space [27]. However, a central goal in immunological studies is to identify specific immune features associated with continuous outcomes such as age or disease progression, and to estimate the direction and magnitude of these associations to better understand cellular function and immune-system dynamics. In this context, an analysis technique that focuses on both variable selection and coefficient estimation is preferred.

For high-dimensional settings, regression techniques, such as the lasso [25] and adaptive lasso [29], have been widely utilized to select important variables and estimate their effects on an outcome by shrinking less important coefficients toward zero, thereby encouraging model sparsity [5, 15, 17]. However, the interdependence of cellular parameters often results in multicollinearity, where predictors are highly correlated and their individual contributions become difficult to distinguish. Lasso has previously been shown to inadequately handle highly correlated parameters, often selecting only one predictor among the correlated ones. To address this, the elastic net [30] and adaptive elastic net [31] combine the lasso penalty with an additional penalty that stabilizes coefficient estimates by shrinking correlated predictors toward similar, smaller values. This combination allows correlated predictors to be retained together while controlling model complexity. Additional methods have since been developed to tackle more complex correlation scenarios (varying magnitude or direction) such as random lasso [26], Hi-LASSO [13], the nonparametric bootstrap quantile method [1], and the Robust Elastic Net via Bootstrap (RENBOOT) [12]. These methods address multicollinearity and overfitting mainly by bootstrap resampling-based aggregation, which stabilizes selection when predictors are highly correlated and reduces variance across samples. However, the nonparametric bootstrap quantile method and RENBOOT focus primarily on variable selection rather than direct coefficient estimation.

**Fig. 1:**
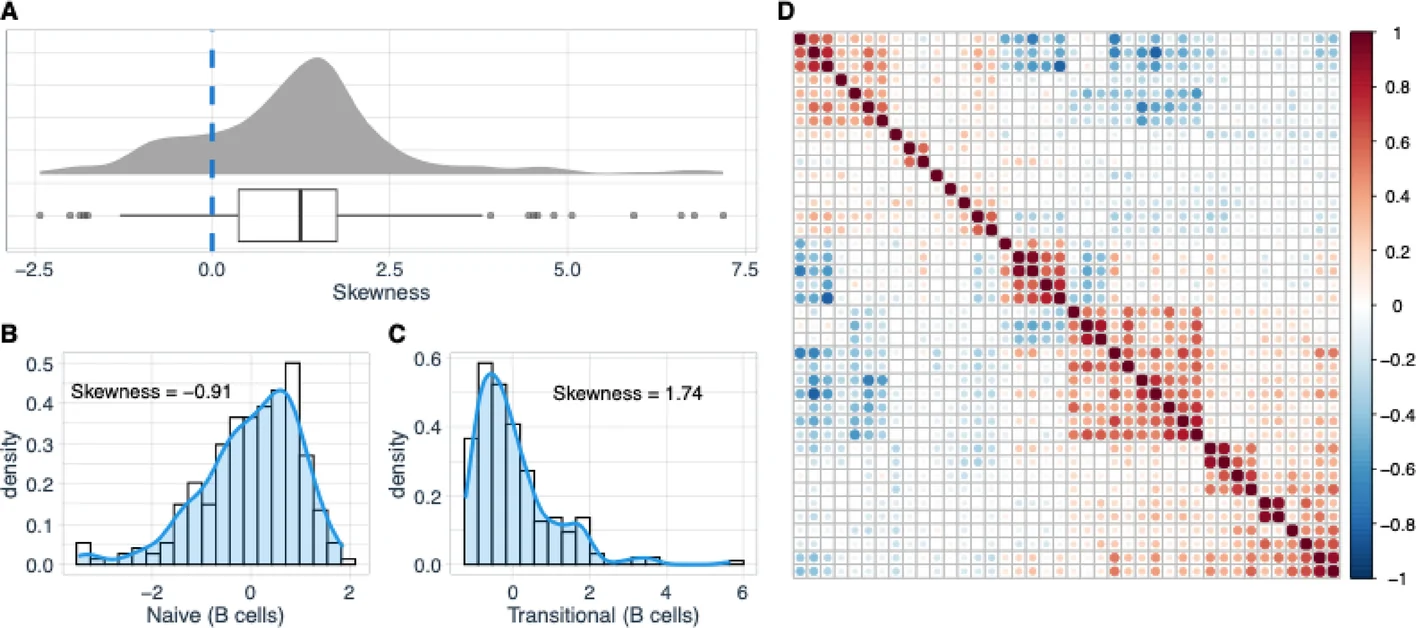
Skewness and correlation in published flow cytometric immunophenotyping data [20]. **A** Distribution of skewness for 192 immunophenotyping variables. Positive skewness indicates a right-skewed distribution, while negative skewness indicates a left-skewed distribution. **B** Representation of negative skewness, exemplified by the z-scored percent of naïve B cells. **C** Representation of positive skewness, exemplified by the z-scored percent of transitional B cells. **D** Correlation matrix for a subset of significantly correlated variables from the immunophenotyping data. Positive correlations are displayed as various shades of red and negative correlations are indicated by shades of blue

Accurate coefficient estimation following variable selection remains a challenging issue in complex multicollinearity scenarios [9]. Immunophenotyping datasets exhibit high multicollinearity and skewed distributions, which can distort standardization and residuals, leading to biased feature selection and unreliable coefficient estimates (Fig. 1A). To address these challenges, we propose a new approach called the Bootstrap-Enhanced Regularization Method (BERM). BERM integrates bootstrapped confidence intervals with penalized regression techniques to obtain robust variable selection and coefficient estimation. In Sect. ''Methods'' we present our methodology and describe how it differs from existing approaches. Then in Sect. "Simulation study", we demonstrate BERM’s performance in simulation studies, where data is simulated with varying degrees of skewness, correlation, and sparsity. In Sect. ''Case study'', we apply our method to an immunophenotyping dataset comprised of 192 flow-cytometry derived phenotypes on peripheral blood mononuclear cells to identify immune features associated with aging and characterize age-related changes in the immune system. A discussion of our findings is presented Sect. "Discussion".

## Methods

### Problem setup and notation

Consider a human immunophenotyping dataset comprising * n* individuals, each characterized by a response variable $\:{\mathrm{y}}_{\mathrm{i}}$ representing immune age, and a corresponding * p*-dimensional predictor vector $\:{\mathrm{x}}_{\mathrm{i}}=({\mathrm{x}}_{\mathrm{i}1},{\mathrm{x}}_{\mathrm{i}2},...,{\mathrm{x}}_{\mathrm{i}\mathrm{p}})$ indicating the immunophenotyping variables. The relationship between the predictors and the response is generally modeled using the linear equation:

1 $$ y_{i} = \beta _{1} x_{{i1}} + \beta _{2} x_{{i2}} + ~ \ldots + \beta _{p} x_{{ip}} + \epsilon _{i} $$

, where $\:{\epsilon}_{\mathrm{i}}$ denotes the error term, assumed to be independently and identically distributed according to a normal distribution with zero mean and constant variance. The immunophenotyping variables are standardized and the immune age is mean-corrected, eliminating the need for an intercept in the equation. Our goal is to identify a significantly associated subset of * p* predictors and to obtain accurate coefficient estimates.

### BERM

We developed a two-step method which we call Bootstrap-Enhanced Regularization Method (BERM), that integrates techniques from both the adaptive elastic net and bootstrapping confidence intervals. In the first step of BERM, an elastic-net based bootstrapping confidence interval is obtained via data resampling. Variables with coefficients within an estimated 95% confidence interval $\:{\widehat{CI}}_{j}=\left[{\widehat{\beta\:}}_{j}^{0.025},{\widehat{\beta\:}}_{j}^{0.975}\right]$ that covers zero are deemed irrelevant. The relevance is directly incorporated into the second step of BERM through variable-specific penalty weights in a weighted elastic net model. The penalty weight, $\:{w}_{j}$, is set to 1 for relevant variables (i.e., $\:0\notin\:{\widehat{CI}}_{j}$) and $\:\infty\:$ for irrelevant variables, so that variables whose estimated bootstrap confidence intervals include zero are excluded from the model. Coefficient estimates are then obtained by minimizing:

2 $$\:\underset{({{\upbeta\:}}_{0},{\upbeta\:})}{\mathrm{min}}\frac{1}{2\mathrm{n}}{\sum\:}_{\mathrm{i}=1}^{\mathrm{n}}{\left({\mathrm{y}}_{\mathrm{i}}-{{\upbeta\:}}_{0}-{\sum\:}_{\mathrm{j}=1}^{\mathrm{p}}{{\upbeta\:}}_{\mathrm{j}}{\mathrm{x}}_{\mathrm{i}\mathrm{j}}\right)}^{2}+{\uplambda\:}{\sum\:}_{\mathrm{j}=1}^{\mathrm{p}}{\mathrm{w}}_{\mathrm{j}}\left({\frac{1-{\upalpha\:}}{2}{\upbeta\:}}_{\mathrm{j}}^{2}+{\upalpha\:}\left|{{\upbeta\:}}_{\mathrm{j}}\right|\right)$$

, where $\:\frac{1-{\upalpha\:}}{2}{\beta\:}_{j}^{2}$ and $\:{\upalpha\:}\left|{\beta\:}_{j}\right|$ represent the shrinkage (L2) and sparsity (L1) penalty components, respectively. BERM is available as an R package on GitHub (https://github.com/xiaorudong/berm).

### Fitting BERM

To optimize the computational efficiency of BERM, we set $\:{\upalpha\:}$ = 0.5 by default, to evenly distribute the influence of the two penalties. Users may optionally tune $\:{\upalpha\:}$ through cross-validation when additional computational resources are available. Bootstrapping utilizes 100 resampling iterations by default. The train () function in the caret R package is used to tune the parameter $\:{\uplambda\:}$ [14]. Using the bootstrap samples, a 95% confidence interval for each coefficient is constructed from the 2.5th and 97.5th percentiles of the bootstrap estimates. In the second step, the penalty.factor argument in the train () function is set to reflect each variable’s relevance determined in the first step, with irrelevant variables assigned Inf excluded from the final model.

### Differences from existing approaches

Existing methods only partially address the joint problem of variable selection and coefficient estimation under severe multicollinearity. Adaptive elastic net [31] and adaptive lasso [29] incorporate weights estimated from an initial fit into the penalty terms, making performance sensitive to that first estimation. Random lasso [26]and Hi- LASSO [13] improve selection stability by subsampling predictors but require additional tuning decisions. RENBOOT [12] uses bootstrap intervals to identify important variables, however does not address coefficient estimation following variable selection. Our approach uses bootstrap confidence-intervals to determine variable inclusion, then applies a weighted elastic-net estimation in a second stage to improve both variable selection and coefficient estimation.

### Simulation study

#### Setup

Considering the dimensionality of realistic immunophenotyping datasets, we simulated data for two dimensionality settings: moderate-dimensional ($\:\mathrm{large}\:n>\mathrm{moderate}\:p$) and high-dimensional ($\:\mathrm{large}\:n\ll\:\mathrm{large}\:p$) spaces. For each setting, the true model was simulated according to three patterns of sparsity (i.e., the true proportion of zero coefficient variables): balanced (50% sparsity), a predominance of zero coefficients (75% sparsity), and a predominance of non-zero coefficients (25% sparsity). Additionally, simulated datasets were generated with varying noise levels, with $\:{\upsigma\:}\in\:\{1,\:3,\:5\}$.

For the moderate-dimensional setting, the datasets consisted of 60 covariates and 300 observations. Non-zero coefficients were generated from $\:{\upbeta\:}\sim\:\:\mathrm{N}(0,\:{4}^{2})$. To simulate datasets with multicollinearity and skewness, the covariate matrix was generated as a multivariate non-normal distribution using the mnonr R package [19]. Specifically, the multivariate skewness was set to 5,000 and multivariate kurtosis to 25,000. Multivariate skewness indicates deviation from symmetry expected under a multivariate normal distribution and multivariate kurtosis contributes to the heaviness of the tails of the distribution in a multivariate space. The covariance matrix for each dataset was set as:

$$\:\left[\begin{array}{ccc}{\sum\:}_{\mathrm{U}(0.6,\:\:1)}^{20}&\:0&\:0\\\:0&\:{\sum\:}_{\mathrm{U}(0.3,\:0.5)}^{20}&\:{\mathrm{J}}_{0.2}^{20}\\\:0&\:{\mathrm{J}}_{0.2}^{20}&\:{\mathrm{J}}_{0.05}^{20}\end{array}\right]$$

, where $\:{\sum\:}_{\mathrm{U}(\mathrm{a},\:\mathrm{b})}^{\mathrm{k}}$ is a $\:\mathrm{k}\times\:\mathrm{k}$ symmetric matrix with unit diagonal elements and off-diagonal elements drawn from a uniform distribution $\:\mathrm{U}(\mathrm{a},\:\:\mathrm{b})$. $\:{\mathrm{J}}_{\mathrm{v}}^{\mathrm{k}}$ is a $\:\mathrm{k}\times\:\mathrm{k}$ matrix with all elements value $\:\mathrm{v}$. For generating covariance matrices indicative of different correlation strengths, $\:{\sum\:}_{\mathrm{U}(0.6,\:1)}^{20}$ was used to reflect high correlation with elements from $\:\mathrm{U}\left(0.6,\:1\right)$, while $\:{\sum\:}_{\mathrm{U}(0.3,\:0.5)}^{20}$ corresponded to moderate correlation with elements from $\:\mathrm{U}\left(0.3,\:0.5\right)$. Additionally, $\:{\mathrm{J}}_{0.05}^{20}$ and $\:{\mathrm{J}}_{0.2}^{20}$ were applied to introduce minimal correlation between variables.

For the high-dimensional setting, the datasets were simulated with 500 covariates and 300 observations. The covariate matrix was similarly generated from a multivariate non-normal distribution, but with a skewness of 10,000 and a kurtosis of 300,000 to ensure deviation from normality. The high-dimensional covariance matrix was generated similarly as before with:

$$\:\left[\begin{array}{cccc}{\sum\:}_{\mathrm{U}(0.6,\:\:1)}^{100}&\:0&\:0&\:0\\\:0&\:{\sum\:}_{\mathrm{U}(0.3,\:\:0.5)}^{100}&\:{\mathrm{J}}_{0.1}^{100}&\:0\\\:0&\:{\mathrm{J}}_{0.1}^{100}&\:{\mathrm{J}}_{0.1}^{100}&\:0\\\:0&\:0&\:0&\:{\mathrm{I}}^{200}\end{array}\right]$$

, where $\:{\mathrm{I}}^{\mathrm{k}}$ is a $\:\mathrm{k}\times\:\mathrm{k}$ matrix with unit diagonal elements and off-diagonal element set to 0. Examples of data generated from these scenarios are illustrated in Supplementary Fig. 1.

#### Simulation of simple datasets

To assess methods performances affected by skewness and correlation specifically, we generated ‘simple’ datasets lacking skewness and correlation under the same simulation settings described above using the rnorm() function with mean zero and standard deviation of one. Examples of data generated from these scenarios are illustrated in Supplementary Fig. 2.

#### Simulation evaluation

The balanced accuracy was used to evaluate the performance of each method on the simulated datasets in terms of variable selection accuracy by providing a balanced assessment of sensitivity and specificity.

3 $$ {\mathrm{Balanced~Accuracy}} = 0.5 \times \left( {\frac{{{\mathrm{TP}}}}{{{\mathrm{TP}} + {\mathrm{FN}}}} + \frac{{{\mathrm{TN}}}}{{{\mathrm{TN}} + {\mathrm{FP}}}}} \right) $$

, where TP (True Positive) represents the number of correctly selected nonzero variables, TN (True Negative) represents the number of correctly non-selected zero variables, FP (False Positive) represents the number of falsely selected zero variables, and FN (False Negative) represents the number of non-selected nonzero variables.

The mean squared error (MSE) was used to assess each method’s performance in terms of coefficient estimation by quantifying the discrepancy between the true and estimated coefficients. MSE was calculated only on the accurately selected variables for each method.

4 $$ MSE = \frac{1}{p}\sum\limits_{{j = 1}}^{p} {\left( {\beta _{j} - \hat{\beta }_{j} } \right)} ^{2} $$

, where $\:{{\upbeta\:}}_{\mathrm{j}}$ represents the true coefficient value and $\:{\widehat{{\upbeta\:}}}_{\mathrm{j}}$ denotes the estimated coefficient value for variable $\:\mathrm{j}$.

Differences in balanced accuracy and MSE between BERM and the pooled set of all other methods were evaluated using two-sided t-tests.

## Simulation results

To understand how BERM and existing methods performed in identifying variables and estimating their effects, we simulated data matching the skewness and multicollinearity characteristics of immunophenotyping data (Supplementary Fig. 1). We also generated data across scenarios varying in dimensionality, noise level, and sparsity to represent a wide range of possibilities in biomedical datasets: moderate-dimensional settings (e.g., 60 variables and 300 observations) and high-dimensional settings (e.g., 500 variables and 300 observations). To account for wide-array of true models, we simulated three patterns of sparsity: balanced (50% sparsity), a predominance of zero coefficients (75% sparsity), and a predominance of non-zero coefficients (25% sparsity).

We compared BERM to traditional penalized models (Lasso, Elastic Net), their extensions (adaptive lasso, adaptive elastic net, RENBOOT), and methods specific to high-multicollinearity (random lasso and Hi-LASSO) (details on setup in Supplementary Methods). Performance on feature selection accuracy was evaluated using balanced accuracy to account for the imbalance of zero and non-zero features. The evaluation of estimation bias was only computed over the accurately predicted variables for each method to distinguish estimation from accuracy in variable selection.

### Accuracy in feature selection across simulation scenarios

**Fig. 2:**
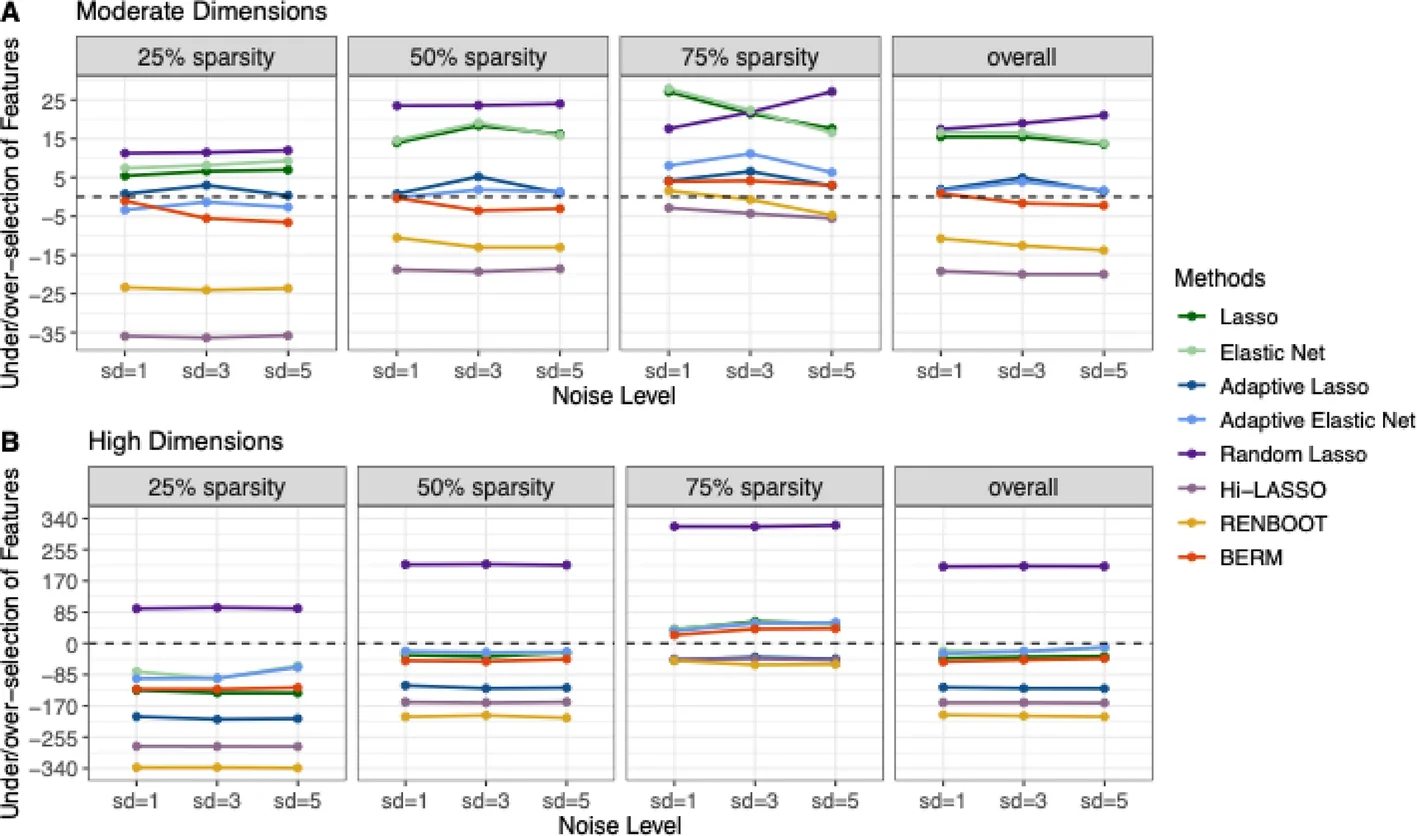
Assessment of under- or over-selection across moderate- and high-dimensional simulation scenarios. **A** The average difference between the number of features selected and the true number of non-zero variables (y-axis) for moderate-dimensional data simulated under various noise levels (x-axis) and model sparsities (panels) is shown for eight different methods. **B** Same as A, but for the high-dimensional scenario

We first compared the degree to which various methods either over- or under-selected features compared to the true number of important variables (sparsity-level). Methods largely differed in their performance across sparsity levels, although all methods tended to select more variables as the model sparsity increased (Fig. 2). In moderate-dimensional settings, the adaptive methods and BERM performed the most consistently in selecting the accurate number of features across various noise- and sparsity levels (Fig. 2A, Supplementary Table 1). Lasso, random lasso, and elastic net consistently over-selected features compared to the true sparsity level, while RENBOOT and Hi-LASSO under-selected features, particularly in low-sparsity scenarios. In the high-dimensional setting, most methods were prone to under-selection in the low- and mid-sparsity scenarios (Fig. 2B, Supplementary Table 2). While most methods had comparable performance between the moderate- and high-dimensional settings, standard elastic net and lasso performed remarkably better in the high-dimensional setting. In contrast, adaptive lasso performed worse in high dimensions, being overly conservative and consistently underestimating the number of relevant features.

**Fig. 3:**
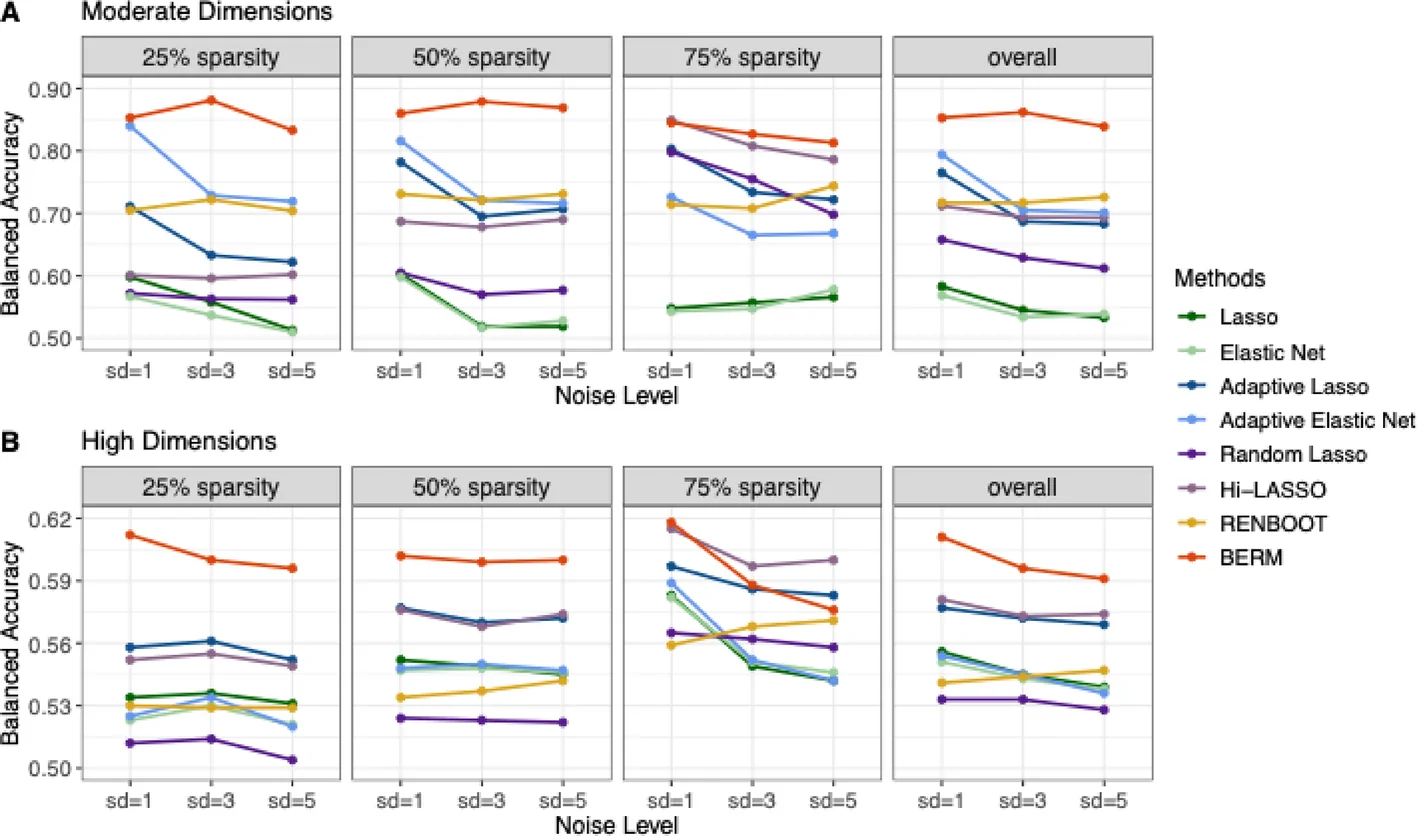
Feature selection accuracy across moderate- and high-dimensional simulated datasets. **A** Mean balanced accuracy of correctly selecting non-zero variables (y-axis) for moderate-dimensional data simulated under various noise levels (x-axes) and model sparsities (panels) is shown for eight different methods. **B** Same as A, but for the high-dimensional scenario

Next, we examined each method’s variable selection accuracy in identifying the truly non-zero features across the simulated scenarios (Fig. 3). Relative to the average balanced accuracy across all scenarios, elastic net, lasso, and random lasso underperformed (ranging from − 7.4% to -3.7% decrease in accuracy), the second-generation penalization methods (e.g. adaptive elastic net, adaptive lasso, RENBOOT and Hi-LASSO) performed slightly above average (ranging from + 1.3% to + 2.3% increase in accuracy), while BERM performed best (+ 10.6% accuracy, *p* = 0.001). BERM had the largest average difference in accuracy between dimensionalities; while still outperforming all other methods in both settings and particularly, in the moderate-dimensionality setting with an average of 17.0% higher accuracy (*p* < 0.001) over the mean performance (Fig. 3A), with the next best performer, adaptive elastic net, having a 5.2% increase (*p* = 0.025). With the exception of RENBOOT, all methods had decreased performance as noise levels increased. Most drops in accuracy across noise levels were small (average − 2.6% accuracy from low to high noise), with adaptive elastic net and adaptive lasso displaying the least stable performances across noise levels (difference of -5.5% and − 4.5%, respectively). The majority of methods were fairly stable across sparsity levels, ranging from − 2.7% to + 2.1% differences in accuracy; however, the three adaptations of lasso all performed substantially better in highly sparse versus lowly sparse scenarios (average of + 6.5% to + 13.3% increase in accuracy).

### Accuracy in coefficient estimation across simulation scenarios

**Fig. 4:**
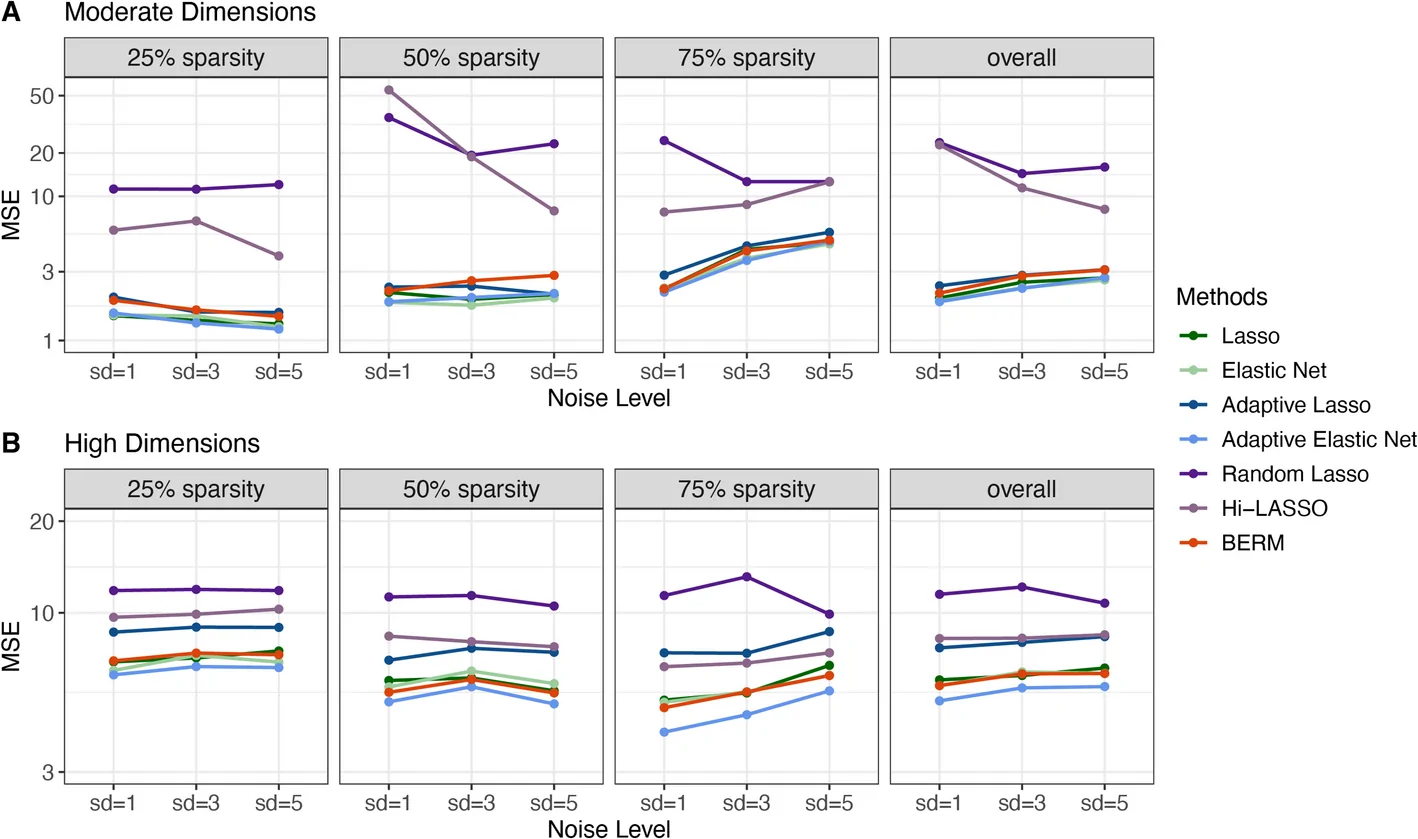
Bias in coefficient estimation across moderate- and high-dimensional simulated datasets. **A** Mean squared error (MSE) was calculated on accurately selected non-zero coefficients (y-axis) for moderate-dimensional data simulated under various noise levels (x-axes) and model sparsities (panels), as shown for seven different methods. **B** Same as A, but for the high-dimensional scenario

The bias between the estimated coefficients and the true simulated coefficient values was calculated only on the accurately selected variables for each method (Fig. 4). BERM showed estimation error comparable to most methods (e.g. adaptive elastic net, elastic net and lasso) across dimensionality settings and varying levels of sparsity and noise (*p* = 0.06–0.99), while consistently yielding lower error than random lasso, and Hi-LASSO. Adaptive lasso had the biggest discrepancy, with an average MSE of 8.0 in the high dimensional setting compared to a MSE of 2.8 in the moderate dimensional setting. Random lasso and Hi-Lasso were also the only two methods to perform better in the high dimensionality setting compared to moderate dimensional, although they had the highest MSE in both settings.

### Influence of skewness and correlation on method performance in feature selection and coefficient estimation

Existing regularization methods are highly sensitive to predictor correlation and skewness. In correlation-only datasets, lasso and elastic net demonstrated limited ability to accurately select relevant features, while adaptive methods (e.g., adaptive lasso and adaptive elastic net) provided modest improvements but remained sensitive to noise and sparsity (Supplementary Figs. 3 & 4). In skewed only datasets, the feature selection performance of lasso and elastic net further deteriorated in moderate-dimensional settings, and regularization methods exhibited substantial variability in coefficient estimation across different noise levels (Supplementary Figs. 5 & 6).

The presence of both correlation and skewness, as observed in immunophenotyping datasets, further challenged existing regularization methods, with feature selection and coefficient estimation performance varying across conditions. We next investigated how these characteristics contributed to each method’s performance. We generated simple simulated datasets similar to the complex simulated data but without skewness and high correlation among the variables. The simulation was evaluated across moderate- and high-dimensional settings along with scenarios differing in sparsity level and noise. We compared the difference in performance for each method and scenario between the simple and complex simulated datasets. Complete results for each scenario are provided in Supplementary Figs. 7 & 8.

**Fig. 5:**
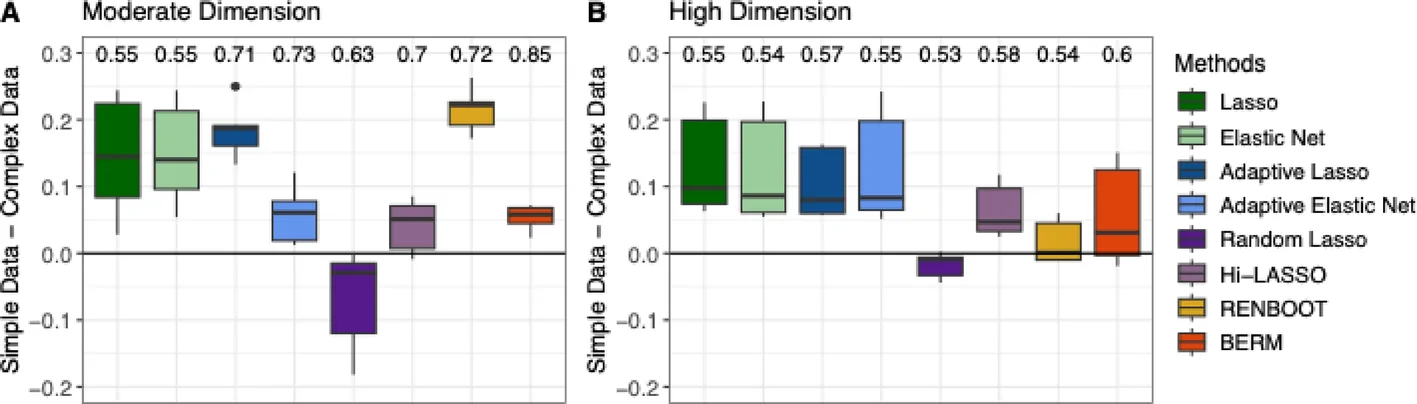
Difference in accuracy of feature selection performance between simple and complex datasets across all scenarios. **A** Mean difference in balanced accuracy between simple and complex datasets of correctly selecting non-zero variables (y-axis) for moderate-dimensional data simulated under various noise levels and model sparsities is shown for eight different methods. The mean balanced accuracy in complex datasets is shown at the top of the figure. **B** Same as A, but for the high-dimensional scenario

In terms of feature selection, all methods except random lasso had better performance in the simple datasets (Fig. 5). BERM tended to have the smallest differences in performance between simple and complex datasets in both moderate- and high-dimensional datasets. In contrast, RENBOOT tended to have the largest differences in performance in the moderate-dimensional setting, where it was the most accurate method for the simple simulated datasets (Fig. 5 and Supplementary Fig. 7). In moderate dimensions, the adaptive methods had variable performance. Adaptive lasso was similarly accurate to BERM and RENBOOT, but had a severe performance drop in complex scenarios, while adaptive elastic net had an average accuracy on simple datasets with only a minor drop in complex datasets (Fig. 5A and Supplementary Fig. 7A). In high-dimensional settings, traditional and adaptive methods performed similarly on simple datasets but experienced declines in performance in complex scenarios. However, random lasso and RENBOOT had minor differences in performance between simple and complex data due to their low performance in both (Fig. 5B and Supplementary Fig. 7B).

**Fig. 6:**
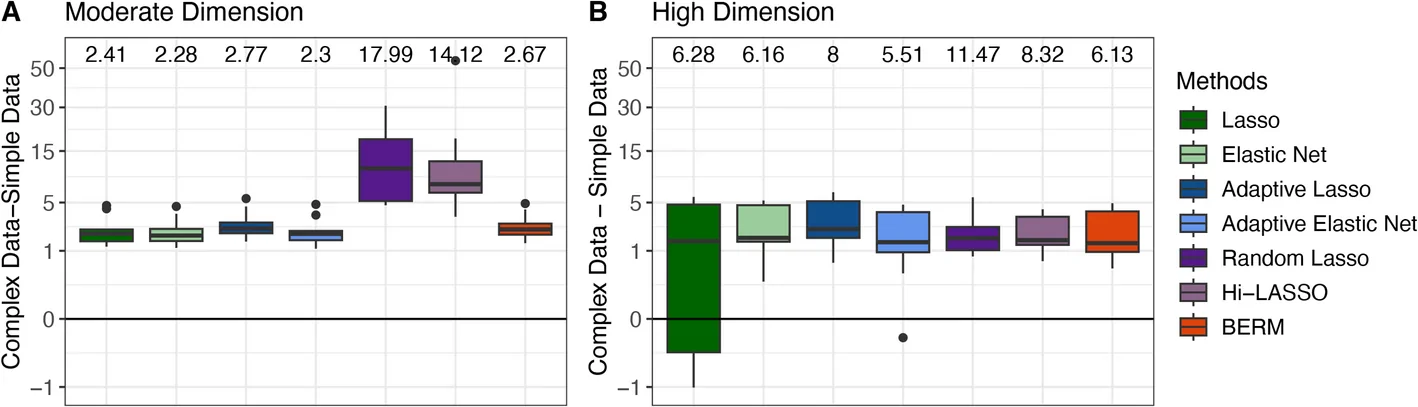
Comparison of mean coefficients estimation between simple and complex datasets across all sparsity and noise levels. **A** Mean difference in MSE between simple and complex datasets for moderate-dimensional data simulated under various noise levels and model sparsities is shown for seven different methods. The mean MSE in complex datasets is shown at the top of the figure. **B** Same as A, but for the high-dimensional scenario

Regarding coefficient estimation on accurately selected variables, the performance on simple datasets was better in the vast majority of cases (Fig. 6). In moderate settings, all methods improved, while in high-dimensional settings, all methods except lasso and adaptive elastic net demonstrated better performance. In the moderate dimensional setting, only Hi-LASSO and random lasso failed to accurately estimate coefficients in simple datasets and were increasingly poor in complex scenarios (Fig. 6A). In the high-dimensional setting, the greatest differences in performance were in the high-sparsity scenario, where the simple datasets had much lower MSE compared to lower sparsity levels (Supplementary Fig. 8B).

## Case study

### Immunophenotyping data

We previously analyzed immunophenotyping data [20] comprised of 192 features derived from spectral flow cytometry on peripheral blood mononuclear cells along with complete blood count (CBC) measurements. Spectral flow cytometry enables simultaneous quantification of a large number of immune cell surface markers, yielding detailed characterizations of immune cell subsets from T cell, B cell, NK cell, and monocyte populations measured from peripheral blood. Because many immunophenotypes represent nested or overlapping cell populations, for example, a parent population and its functional subsets, the features are naturally interdependent and often highly correlated with one another. BERM’s two-step bootstrapped-stabilized approach is well-suited to handle this high dimensionality and multicollinearity.

The cohort included 252 samples in a control group (CTR), defined as healthy individuals without diabetes who tested negative for type 1 diabetes-associated islet autoantibodies; 310 unaffected first-degree relatives of individuals with Type 1 Diabetes (REL), who may carry genetic or environmental predispositions related to the disease yet show no clinical signs of diabetes; and 240 participants diagnosed with Type 1 Diabetes (T1D). We applied BERM to the CTR group to identify features associated with immune aging, with chronological age as the response and all features as predictors (see Supplementary Methods for additional details). The model trained in the CTR group was subsequently applied to the T1D and REL groups, aiming to identify alterations caused by T1D and to further investigate the impact of T1D on the immune system aging.

### Results of immune age model

We applied BERM to the control group of the immunophenotyping dataset aiming to identify features associated with immune aging in the general population. BERM identified 84 features, achieving a test set prediction performance of $\:{\mathrm{R}}^{2}=0.79$, compared to the original analysis (which utilized random lasso) that identified 69 features with a test set prediction performance of $\:{\mathrm{R}}^{2}=0.70$ [20].

When using BERM to predict immune age in the T1D group, we observed that participants younger than 30 years with T1D exhibited significantly accelerated immune aging compared to the CTR and REL groups, with an average increase of 4.47 years (*P* < 0.001, Supplementary Fig. 9). This represents a greater acceleration compared to our previously reported results, in which the average increase was 3.36 years (*P* < 0.001) [20].

There were 45 features selected by both approaches, which we further compared the skewness, correlation relationships, and estimated coefficients of shared and uniquely-identified features. BERM uniquely identified variables with extreme skewness (Fig. 6A). Both methods were effective in identifying highly correlated features and some features uniquely identified by either BERM or random lasso exhibited high correlation with shared selected features (Fig. 6B), whereas features uniquely selected by random lasso were more likely to correlate with those selected by both methods (Fig. 6C). All 45 features identified by both methods showed consistent directions in their coefficient estimates (Supplementary Fig. 10). However, BERM was able to identify variables with subtle effects on immune aging, whereas random lasso, which employs a cutoff for feature selection, failed to recognize these variables with small magnitudes. Several of the 39 features identified exclusively by BERM have direct support in the aging literature. Among immune phenotypes, HLA-DR+ Memory Th1 and Th1/17 cells have been shown to accumulate with age in healthy blood [4, 10, 22, 23]. BERM also uniquely identified CBC-derived parameters in the immunophenotyping dataset, including total WBC, monocyte, and RBC counts, which decline progressively with age [2, 16, 21].

**Fig. 7:**
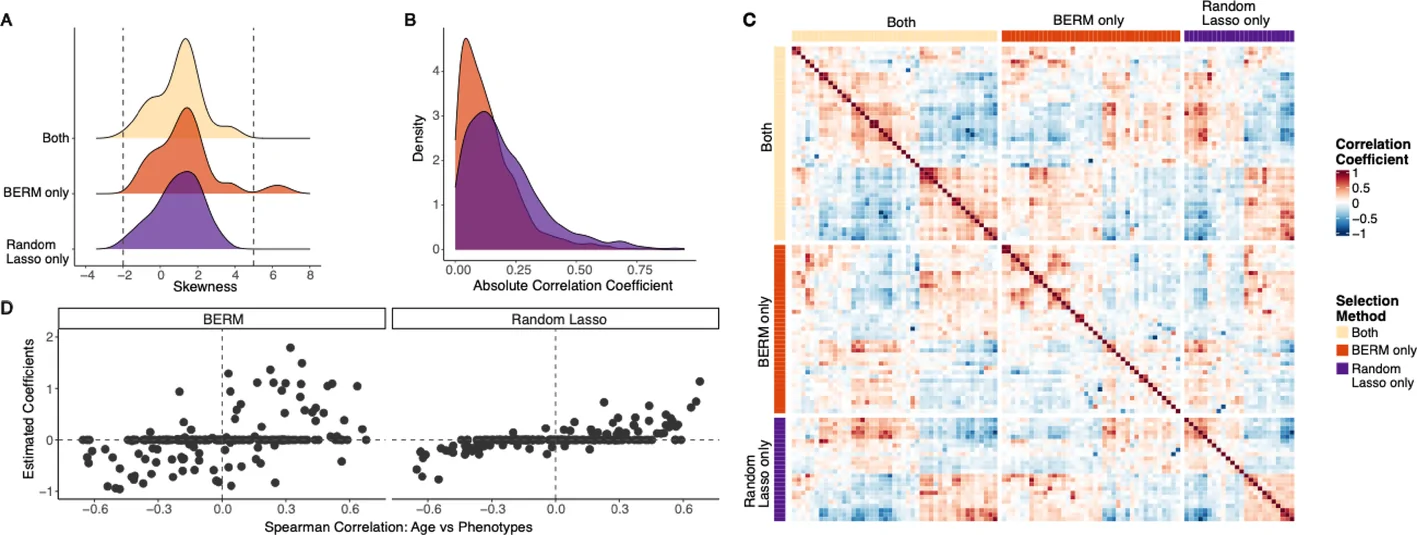
Comparison of feature selection and coefficient estimation by random lasso and BERM in the immunophenotyping dataset [20]. A total of 45 immunophenotyping features were selected by both methods (noted as “Both”), while 39 features were identified exclusively by BERM (noted as “BERM only”), and 24 features were unique to random Lasso (noted as “Random Lasso only”). **A** Skewness of selected features; **B** Absolute value of Spearman correlation coefficients between features selected by both methods and those selected only by random lasso or BERM; **C** Spearman correlation coefficients of selected features; **D** Relationship between the estimated coefficients, as determined by BERM and random lasso, and their correlation with age

To further assess the relevance of the selected variables, we examined their association with immune age. In the CTR group, the estimated coefficients for each immunophenotype from random lasso showed a positive correlation with their respective correlations to age, whereas BERM’s coefficients did not exhibit as strong of a relationship (Fig. 6D). This highlights key differences between the two approaches. Random lasso favored age-correlated predictors, as they consistently received larger coefficients across predictor subset bootstraps, making their final coefficients more reflective of univariate age associations. In contrast, BERM optimizes prediction by selecting variables and weighting them collectively in an elastic net. BERM’s coefficients reflect feature importance within the model, and do not necessarily indicate univariate directional relationships with the outcome.

## Discussion

In the simulation studies, BERM accurately selected important variables and estimated coefficients without clear overfitting. We observed that BERM had the most consistent performance across a wide range of scenarios in both moderate-dimensional and high-dimensional settings. Classical methods lasso and elastic net performed poorly in feature selection for moderate-dimensional datasets, often selecting an excessive number of features—on average, 15 and 16 more, respectively. Additionally, classical methods and their adaptations, including lasso, elastic net, adaptive lasso, and adaptive elastic net, were heavily influenced by complex data structures. Among them, adaptive elastic net was particularly sensitive to noise and sparsity levels, which suggests a propensity to fit to noise rather than to the underlying signal. Random lasso and Hi-LASSO performed well only within a limited range of simulation scenarios, particularly in highly sparse settings with low noise.

RENBOOT shares a similar bootstrap-based variable-selection strategy with the first step of BERM, while additionally incorporating min-max scaling and bias-corrected accelerated bootstrap confidence intervals. It appears most suitable for relatively simple data settings, particularly when predictors are not strongly skewed. Across our simulations, RENBOOT showed strong feature-selection performance in non-skewed scenarios, achieving top performance in moderate-dimensional simple settings and in high-dimensional correlation-only settings with high sparsity. Its use of min-max scaling and bias-corrected accelerated bootstrap intervals may help stabilize variability in bootstrap coefficient estimates; however, in skewed data this stabilization appears to come at the cost of increased conservatism and reduced sensitivity to weak signals. Therefore, RENBOOT may be recommended when the primary goal is reliable variable selection under non-skewed conditions, while caution is warranted in skewed settings where weaker signals may be missed.

BERM takes advantage of the superior performance of the elastic net in handling high multicollinearity and improves upon robustness via bootstrap confidence intervals to significantly improve variable selection in complex data scenarios. On correctly selected variables, BERM’s estimation of coefficients is comparable to that of lasso and elastic net approaches, allowing for accurate prediction. Although BERM incurs a higher computational burden compared to conventional methods like lasso and elastic net due to the use of cross-validation to tune optimal parameter $\:{\uplambda\:}$, it is more efficient than alternatives such as random lasso and Hi-LASSO (Supplementary Table 3). We noted that BERM had reduced feature selection accuracy in extreme scenarios characterized by variables with either all zero coefficients or all non-zero coefficients (Supplementary Table 4). We found that tuning $\:{\upalpha\:}$ via cross-validation rather than using the default value of $\:{\upalpha\:}=0.5$ in BERM’s initial step, enhanced feature selection performance in these challenging scenarios. Users may therefore consider tuning $\:{\upalpha\:}$ when analyzing datasets with expected extreme sparsity structure or when computational resources are not a limiting concern.

An additional limitation of BERM in practice is that it is based on the elastic-net framework, so it assumes that effects are mostly linear and additive. As a result, interaction effects and nonlinear relationships will not be captured unless they are explicitly included in the model. Evaluating BERM under these structures is an important direction for future research. Additionally, the bootstrap-based relevance step may be less stable in small samples. This could lead to the exclusion of predictors that have weak effects.

Applied to the immunophenotyping dataset, BERM demonstrated superior predictive capabilities for estimating immune age. While random lasso provided biologically intuitive coefficients that reflected univariate age relationships, BERM’s ensemble approach captured more complex, multivariable predictive patterns and a broader range of features. This expanded feature capture comes with the caveat that some BERM-unique features may reflect cohort-specific variation rather than universally generalizable biology; their individual relevance should be confirmed in independent datasets before mechanistic interpretation.

BERM’s ability to balance feature selection and predictive accuracy makes it particularly valuable in biomedical data analyses, where the goal is to obtain both a sparse model of important features along with an accurate prediction model. Additionally, given the uncertainty of the true sparsity model and noise-level in any data analysis, it is essential to have a method that delivers reliable performance across different scenarios. BERM outperformed all methods for feature selection across a wide-variety of scenarios and demonstrated accuracy in coefficient estimation comparable to current leading approaches. These results highlight the potential of BERM as a robust tool for analyzing immunophenotyping data or similarly complex biomedical data.

## Conclusions

BERM is a two-step regularization approach that advances beyond existing bootstrap-based methods by jointly addressing variable selection and coefficient estimation in complex biomedical datasets. While existing immunophenotyping analysis methods are largely focused on classification or dimension reduction, BERM addresses a distinct goal of identifying which specific features are associated with a continuous outcome and reliably estimating the direction and magnitude of those associations. Compared to traditional regularization approaches, BERM overcomes challenges imposed by high multicollinearity and skewed distributions to yield more reliable variable selection and coefficient estimates. Applied to a 192-parameter human immunophenotyping dataset, BERM captured biologically supported immune aging features, including those with subtle effects and extreme skewness. These findings demonstrate BERM’s potential to support interpretable, quantitative characterization of immune-system dynamics in high-dimensional biomedical research.

## Supplementary Information

Below is the link to the electronic supplementary material.

Supplementary Material 1.

Supplementary Material 2.

## Data Availability

The analyzed immunophenotyping data is available as a supplement in Shapiro et al., 2023. Code for the BERM methodology is available on GitHub at: [https://github.com/xiaorudong/berm](https:/github.com/xiaorudong/berm).

## Abbreviations

BERM: Bootstrap-enhanced regularization method
CRT: Healthy controls
FN: False negative
FP: False positive
MSE: Mean squared error
REL: First-degree relatives of individuals with type 1 diabetes
RENBOOT: Robust elastic net via Bootstrap
T1D: Type 1 diabetes
TN: True negative
TP: True positive
