# Supplementary material for: Bootstrap-enhanced regularization addressing multicollinearity and skewness in high-dimensional immunophenotyping data
Source: BMC Bioinformatics. 2026 Aug 6;27:259. doi: 10.1186/s12859-026-06586-x (PMC13729485; doi:10.1186/s12859-026-06586-x)
Supplement: Supplementary file 1 [file 12859_2026_6586_MOESM1_ESM.pdf]

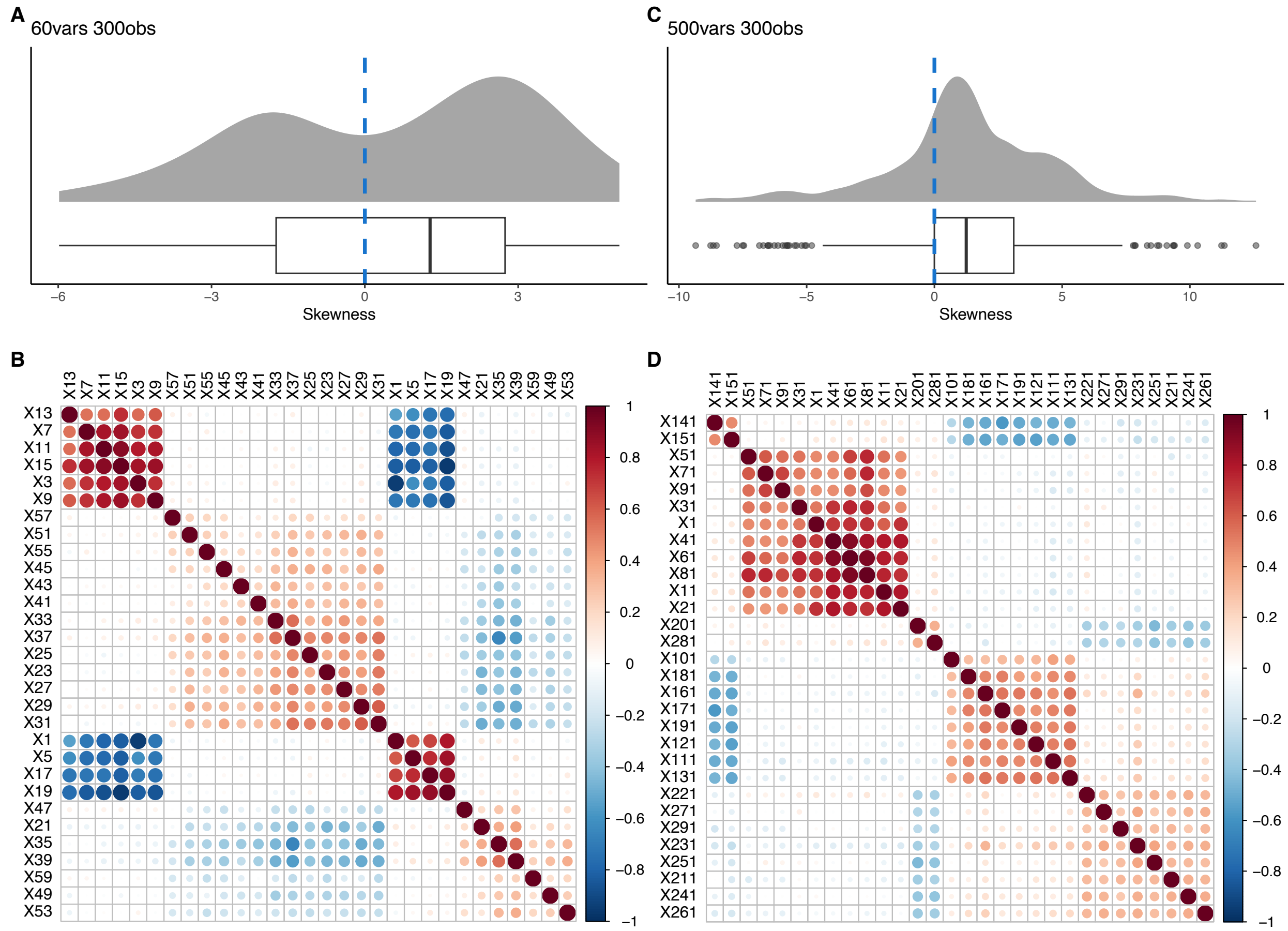

Supplementary Figure 1. Visualization of skewness and correlation in two simulated complex datasets. Visualization of skewness and correlation in two simulated datasets. A, C) Density plots and boxplots of the skewness distribution for variables in a simulated moderate and high-dimensional dataset, respectively. B, D) Correlation matrix for a subset of variables from the simulated moderate and high-dimensional datasets. There are varying degrees of correlation, including low, moderate, and high, as well as a mix of negative and positive correlations.

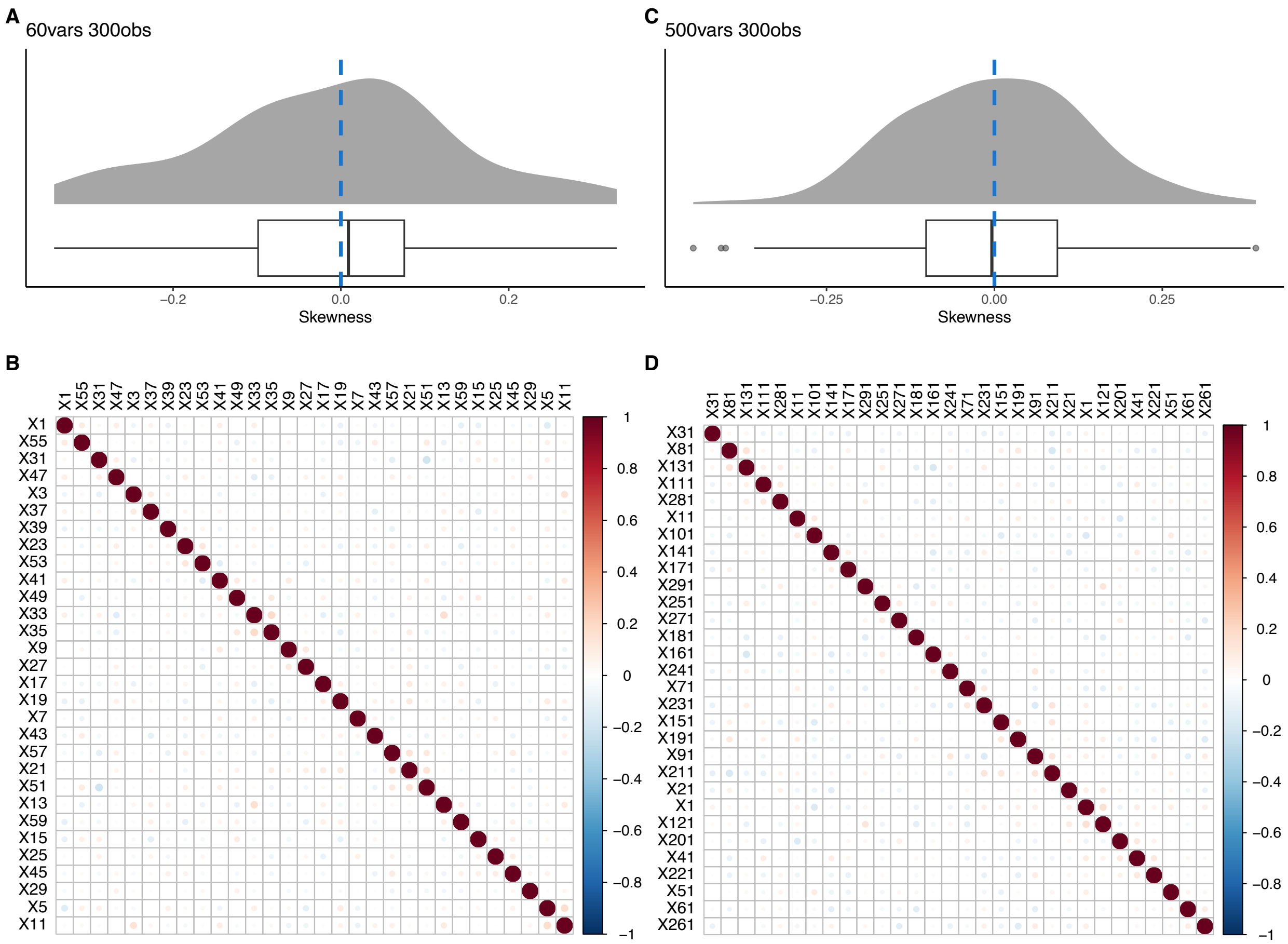

Supplementary Figure 2. Visualization of skewness and correlation in two simple datasets. Visualization of skewness and correlation in two simple datasets. A, C) Density plots and boxplots of the skewness distribution for variables in a simulated moderate and high-dimensional dataset, respectively. B, D) Correlation matrix for a subset of variables from the simulated moderate and high-dimensional datasets. There is no discernible skewness trend or clear correlation between variables.

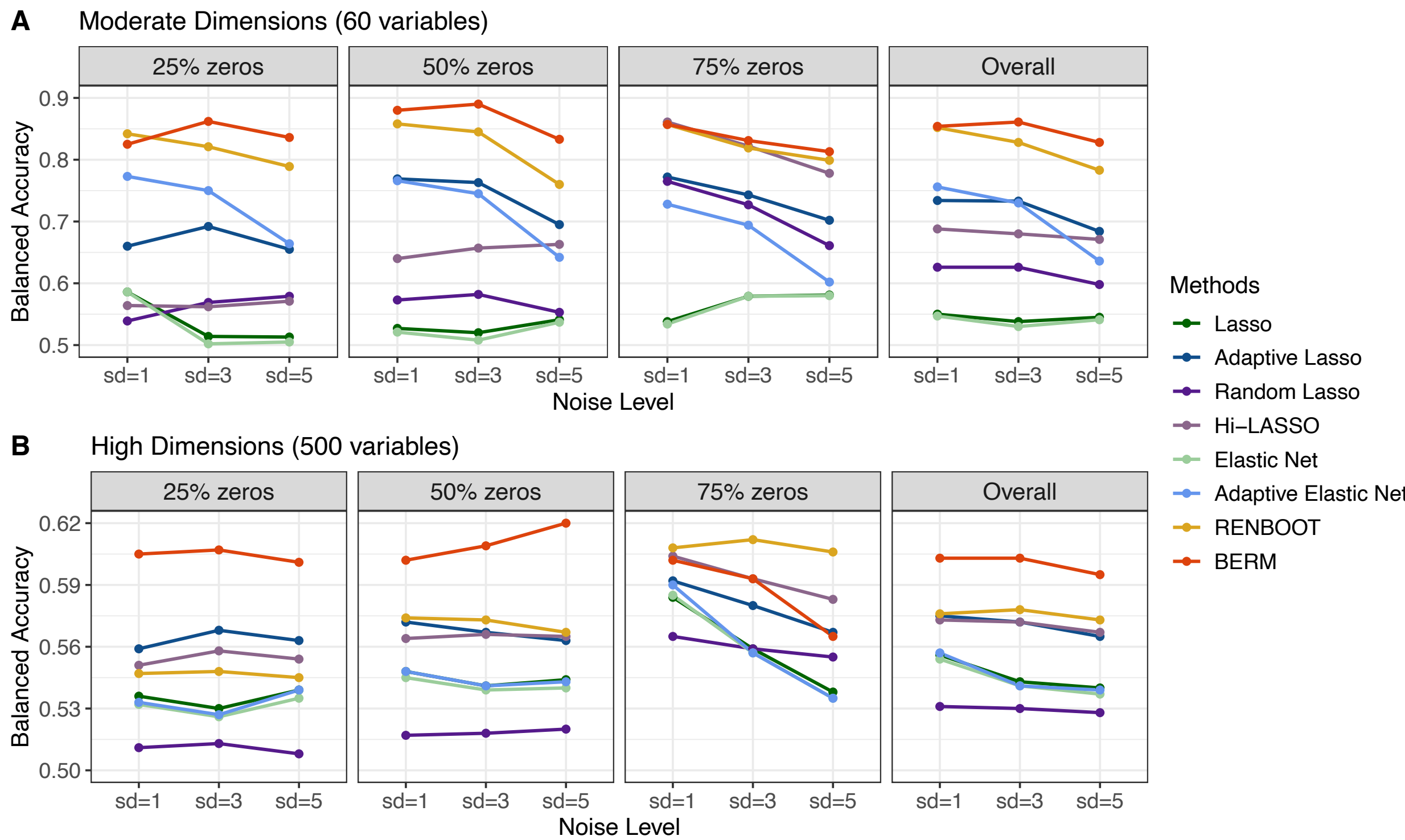

Supplementary Figure 3. Feature selection accuracy across moderate- and high-dimensional simulated *correlation only* datasets. A. Mean balanced accuracy of correctly selecting non-zero variables (y-axis) for moderate-dimensional data simulated under various noise levels (x-axes) and model sparsities (panels) is shown for eight different methods. B. Similar to A for the high dimension scenario.

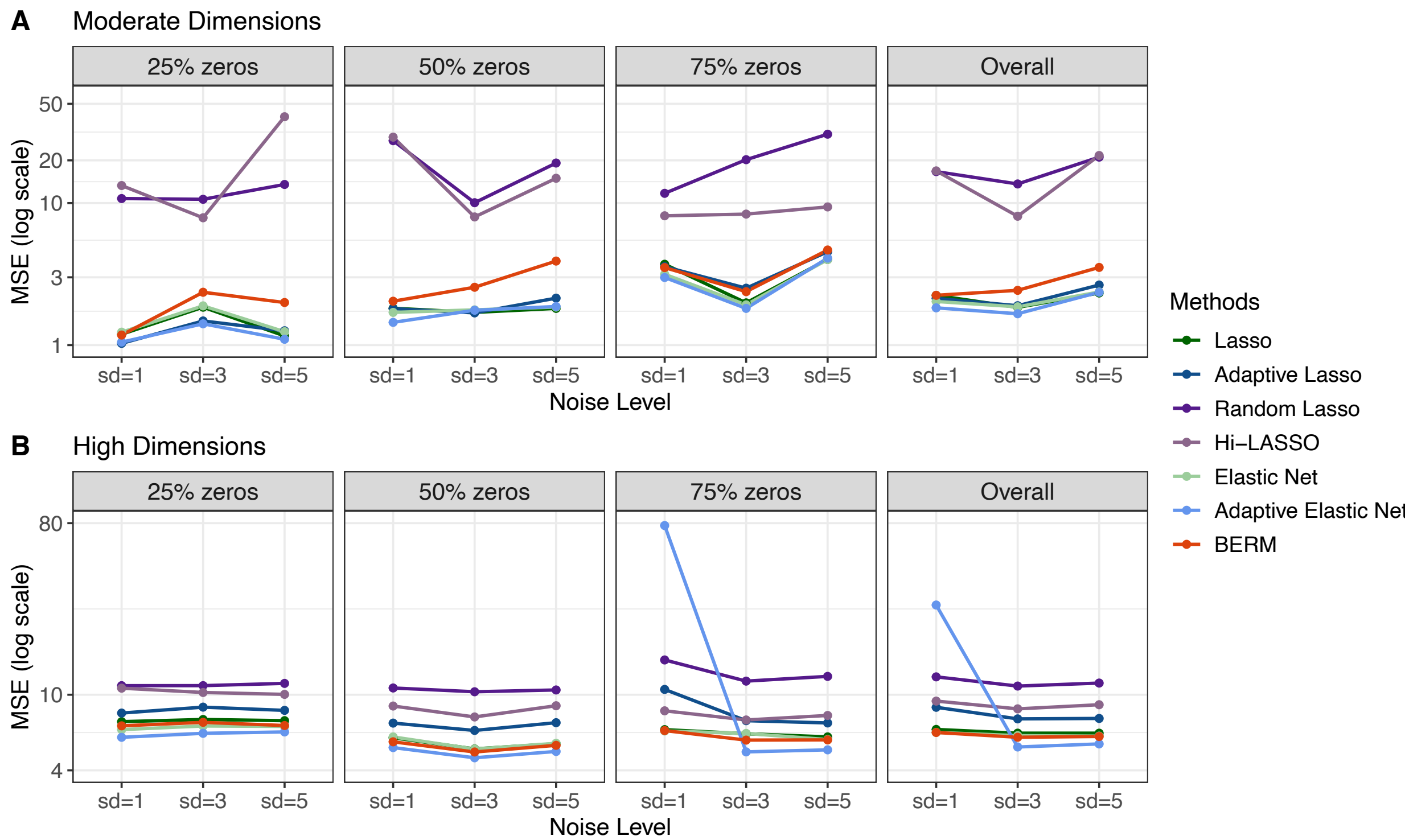

Supplementary Figure 4. Bias in coefficient estimation across moderate- and high-dimensional simulated *correlation only* datasets. A. Mean squared error (MSE) was calculated on accurately selected non-zero coefficients (y-axis) for moderate-dimensional data simulated under various noise levels (x-axes) and model sparsities (panels), as shown for seven different methods. B. Similar to A for the high-dimension scenario.

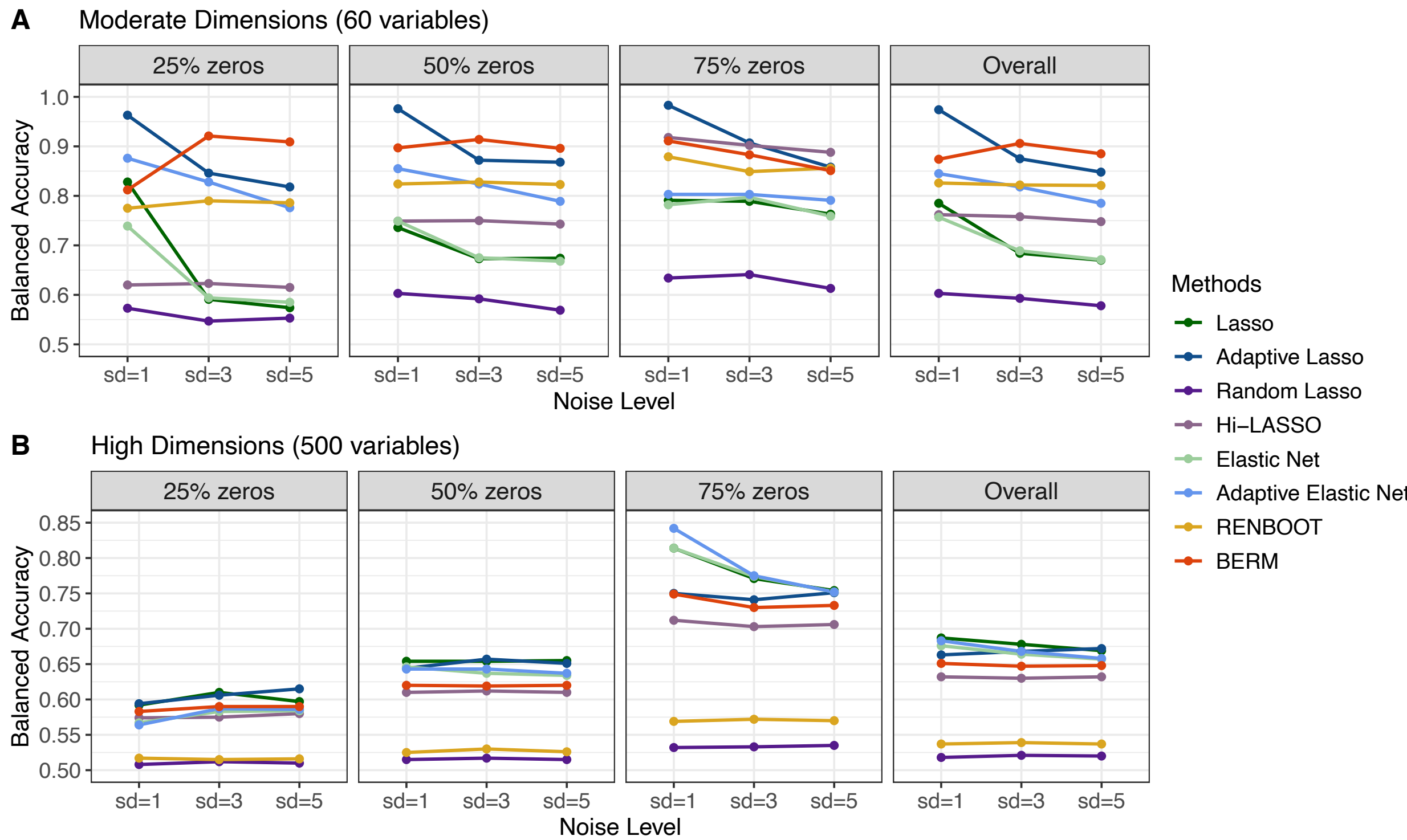

Supplementary Figure 5. Feature selection accuracy across moderate- and high-dimensional simulated *skewness only* datasets. A. Mean balanced accuracy of correctly selecting non-zero variables (y-axis) for moderate-dimensional data simulated under various noise levels (x-axes) and model sparsities (panels) is shown for eight different methods. B. Similar to A for the high dimension scenario.

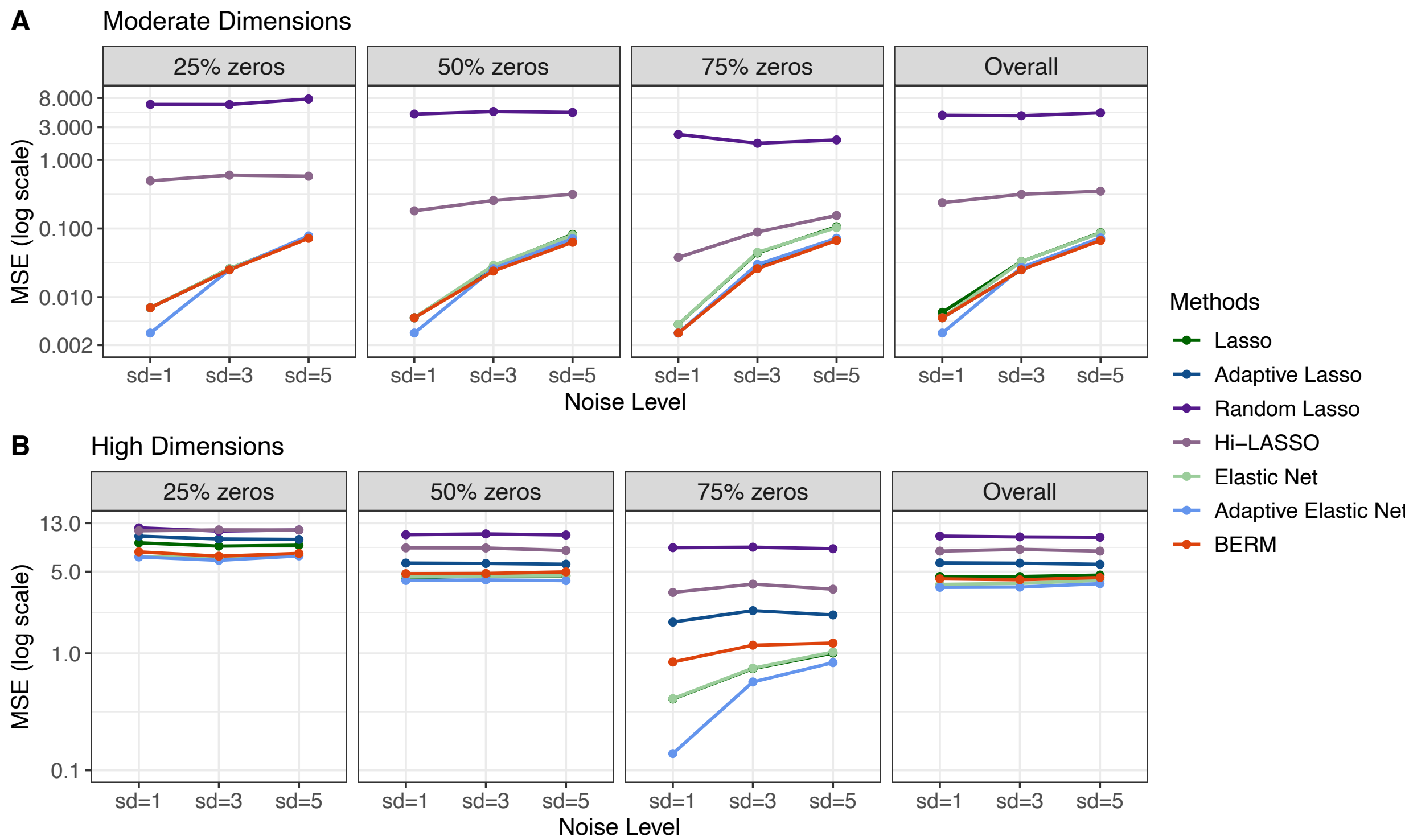

Supplementary Figure 6. Bias in coefficient estimation across moderate- and high-dimensional simulated *skewness only* datasets. A. Mean squared error (MSE) was calculated on accurately selected non-zero coefficients (y-axis) for moderate-dimensional data simulated under various noise levels (x-axes) and model sparsities (panels), as shown for seven different methods. B. Similar to A for the high-dimension scenario.

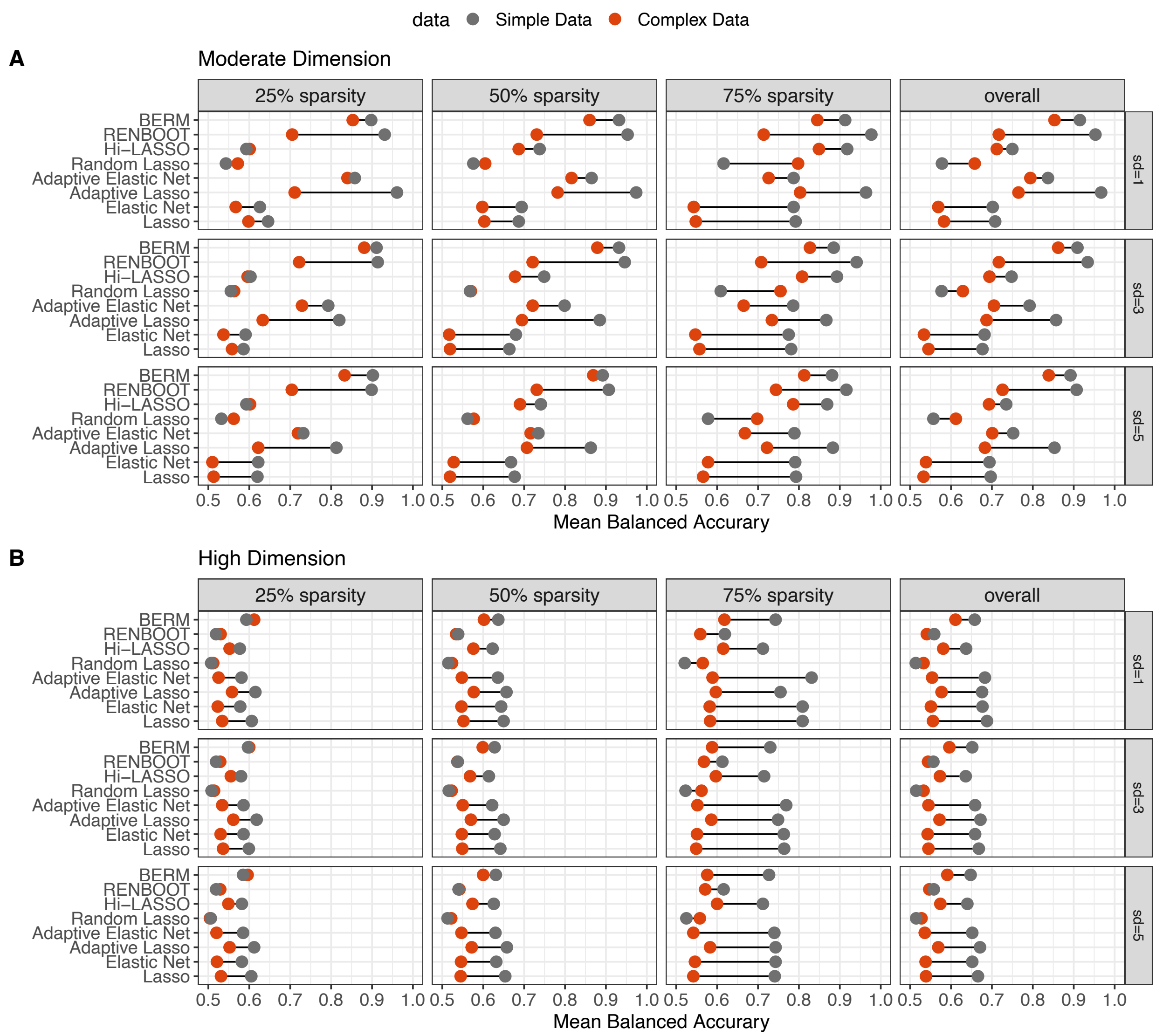

Supplementary Figure 7. Comparison of feature selection performance between simple and complex datasets. This figure illustrates the balanced accuracy of methods evaluated in both moderate- and high-dimensional settings. A) displays results for moderate-dimensional settings, while B) shows results for high-dimensional settings. The comparisons are made between simple datasets, which lack skewness and correlation, and complex datasets that exhibit these complexities. Each panel represents different noise levels (SD=1, SD=3, SD=5) and proportions of zero (25%, 50%, 75%). Colored dots denote the balanced accuracy measurements: one color for the simple datasets and another for the complex datasets. The distance between two corresponding dots within each method and scenario indicates the accuracy difference. A star denotes a significant difference between simple and complex datasets. The Y-axis quantifies the balanced accuracy, while the X-axis categorizes the data by the proportion of zero coefficients.

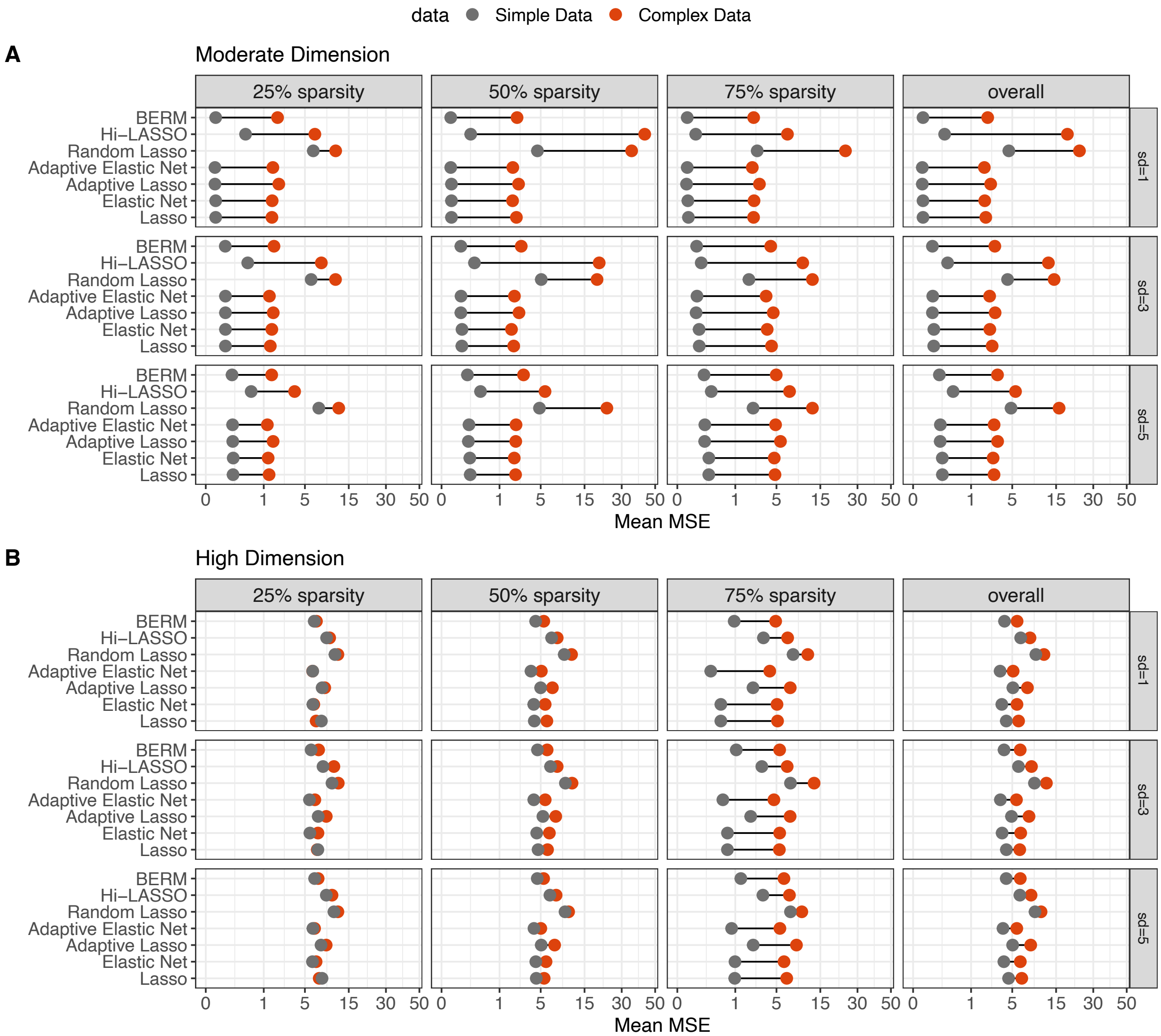

Supplementary Figure 8. Comparison of coefficients estimation performance between simple and complex datasets. This figure illustrates the MSE of methods evaluated in both moderate- and high-dimensional settings. A) displays results for moderate-dimensional settings, while B) shows results for high-dimensional settings. The comparisons are made between simple datasets, which lack skewness and correlation, and complex datasets that exhibit these complexities. Each panel represents different noise levels (SD=1, SD=3, SD=5) and proportions of zero (25%, 50%, 75%). Colored dots denote the balanced accuracy measurements: one color for the simple datasets and another for the complex datasets. The distance between two corresponding dots within each method and scenario indicates the accuracy difference. The Y-axis quantifies the balanced accuracy, while the X-axis categorizes the data by the proportion of zero coefficients.

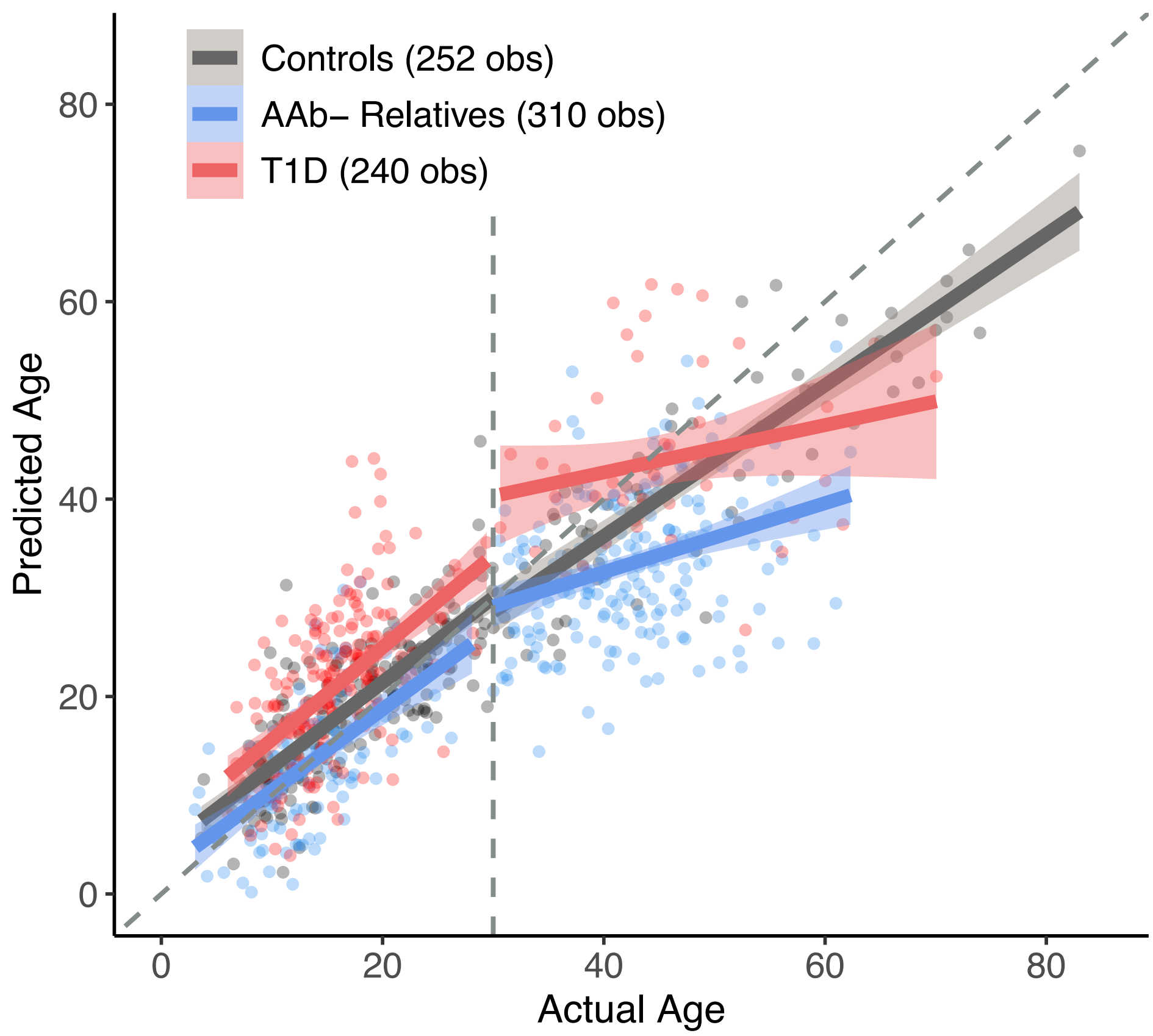

Supplementary Figure 9. Immunophenotype age modeling. BERM was used to estimate immunological predicted age in CTR (gray), T1D (red), and REL (blue). The correspondence of predicted age with chronological age is shown using a piece-wise regression model with a break at chronological age 30.

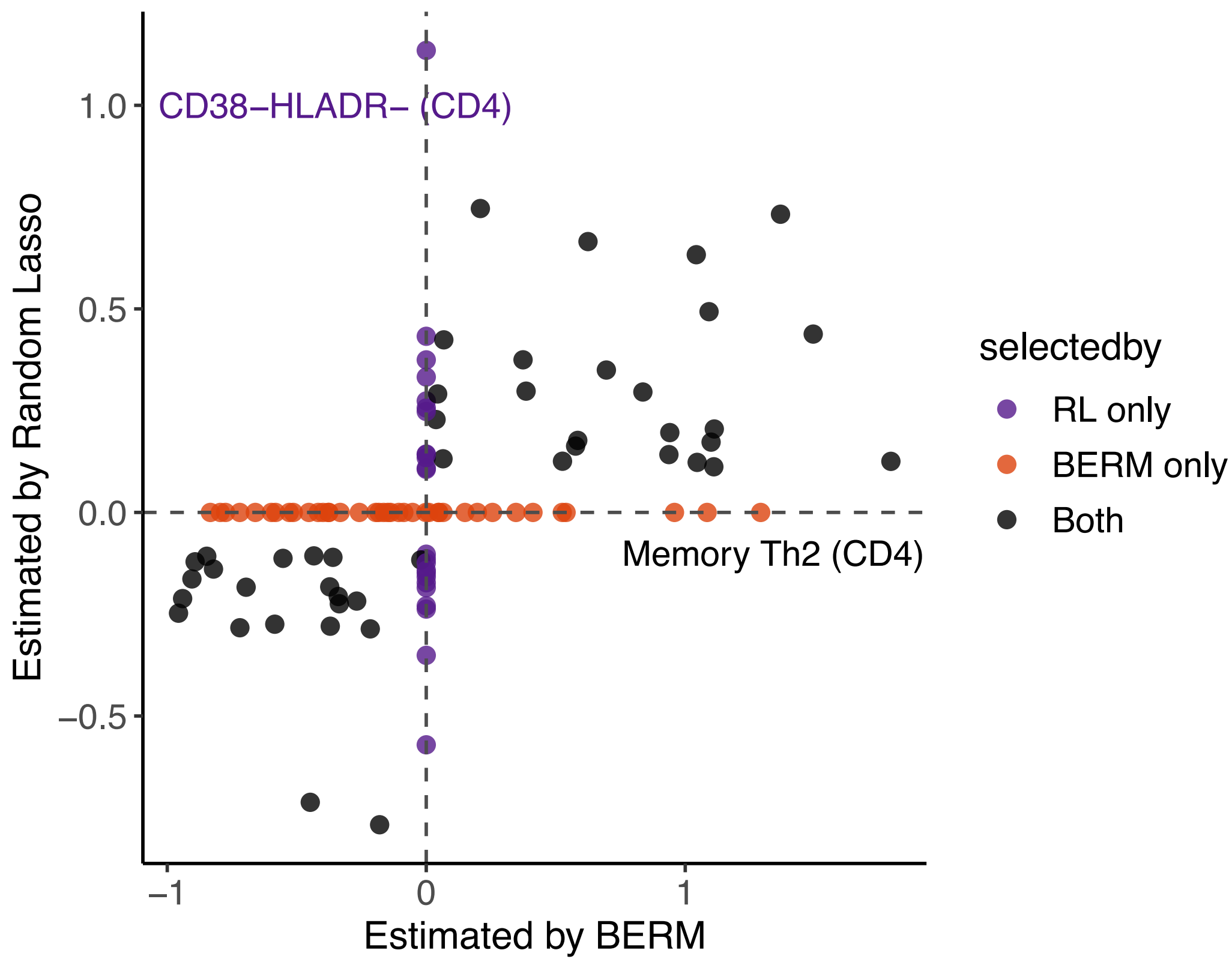

Supplementary Figure 10. Comparison of coefficient estimation by random lasso and BERM in the immunophenotyping dataset (Shapiro et al., 2023). A total of 44 immunophenotyping features were selected by both methods (noted as "Both"), while 46 features were identified exclusively by BERM (noted as "BERM only"), and 25 features were unique to random lasso (noted as "Random Lasso only").

Supplementary Table 1. Performance comparison of BERM and existing methods in moderate-dimensional simulation scenarios under varying conditions of sparsity and noise. Results are evaluated across three criteria: feature selection accuracy, coefficient estimation, and the extent of under- and over-selection. The values before the parentheses represent the mean results across the 20 simulated datasets, while the values within parentheses indicate the standard deviations of these results.

Method	Zero Proportion	Error Noise Level	Balanced Accuracy	MSE	Number of Selected Vars
Lasso	25%zero	sd=1	0.598 (0.078)	1.486 (1.512)	50.4 (3.152)
Lasso	25%zero	sd=3	0.558 (0.064)	1.379 (1.843)	51.6 (3.719)
Lasso	25%zero	sd=5	0.513 (0.05)	1.303 (0.83)	51.95 (2.743)
Lasso	50%zero	sd=1	0.603 (0.105)	2.153 (2.175)	43.95 (7.77)
Lasso	50%zero	sd=3	0.519 (0.075)	1.93 (2.074)	48.35 (5.122)
Lasso	50%zero	sd=5	0.519 (0.064)	2.082 (1.829)	46.15 (6.499)
Lasso	75%zero	sd=1	0.548 (0.062)	2.274 (2.175)	42.1 (5.857)
Lasso	75%zero	sd=3	0.557 (0.074)	4.291 (5.672)	36.45 (6.37)
Lasso	75%zero	sd=5	0.566 (0.093)	4.781 (4.207)	32.65 (12.462)
Lasso	overall	sd=1	0.583 (0.086)	1.971 (1.976)	45.483 (6.823)
Lasso	overall	sd=3	0.545 (0.072)	2.534 (3.803)	45.467 (8.313)
Lasso	overall	sd=5	0.533 (0.074)	2.722 (3.042)	43.583 (11.512)
Adaptive lasso	25%zero	sd=1	0.711 (0.138)	1.999 (1.899)	45.75 (6.648)
Adaptive lasso	25%zero	sd=3	0.633 (0.138)	1.577 (1.981)	48 (5.84)
Adaptive lasso	25%zero	sd=5	0.622 (0.1)	1.566 (1.222)	45.35 (6.063)
Adaptive lasso	50%zero	sd=1	0.782 (0.099)	2.345 (2.47)	30.8 (8.788)
Adaptive lasso	50%zero	sd=3	0.695 (0.097)	2.383 (1.737)	35.2 (7.105)
Adaptive lasso	50%zero	sd=5	0.707 (0.079)	2.097 (1.513)	31 (6.026)
Adaptive lasso	75%zero	sd=1	0.803 (0.078)	2.841 (2.811)	19.1 (7.29)

Adaptive lasso	75%zero	sd=3	0.734 (0.077)	4.532 (6.588)	21.55 (6.039)
Adaptive lasso	75%zero	sd=5	0.722 (0.082)	5.631 (6.552)	17.85 (6.784)
Adaptive lasso	overall	sd=1	0.765 (0.113)	2.395 (2.407)	31.883 (13.311)
Adaptive lasso	overall	sd=3	0.687 (0.113)	2.831 (4.218)	34.917 (12.554)
Adaptive lasso	overall	sd=5	0.683 (0.097)	3.098 (4.284)	31.4 (12.908)
Random lasso	25%zero	sd=1	0.572 (0.067)	11.245 (5.026)	56.25 (2.489)
Random lasso	25%zero	sd=3	0.563 (0.062)	11.207 (4.795)	56.45 (1.905)
Random lasso	25%zero	sd=5	0.562 (0.053)	12.066 (9.436)	57 (2.026)
Random lasso	50%zero	sd=1	0.605 (0.053)	35.231 (69.998)	53.5 (3.269)
Random lasso	50%zero	sd=3	0.57 (0.064)	19.296 (27.346)	53.6 (4.16)
Random lasso	50%zero	sd=5	0.577 (0.063)	23.182 (27.08)	54 (3.509)
Random lasso	75%zero	sd=1	0.798 (0.065)	24.382 (54.157)	32.6 (6.261)
Random lasso	75%zero	sd=3	0.755 (0.042)	12.657 (12.496)	36.85 (3.76)
Random lasso	75%zero	sd=5	0.698 (0.056)	12.658 (12.828)	42.15 (5.06)
Random lasso	overall	sd=1	0.658 (0.118)	23.62 (51.267)	47.45 (11.466)
Random lasso	overall	sd=3	0.629 (0.106)	14.386 (17.638)	48.967 (9.345)
Random lasso	overall	sd=5	0.612 (0.084)	15.969 (18.556)	51.05 (7.439)
Hi-LASSO	25%zero	sd=1	0.601 (0.03)	5.824 (8.119)	9.05 (2.685)
Hi-LASSO	25%zero	sd=3	0.596 (0.029)	6.753 (7.964)	8.6 (2.583)
Hi-LASSO	25%zero	sd=5	0.602 (0.019)	3.861 (5.453)	9.15 (1.694)
Hi-LASSO	50%zero	sd=1	0.689 (0.029)	54.709 (135.91)	11.35 (1.725)
Hi-LASSO	50%zero	sd=3	0.68 (0.034)	18.821 (29.74)	10.8 (2.067)
Hi-LASSO	50%zero	sd=5	0.688 (0.039)	7.93 (8.077)	11.25 (2.359)

Hi-LASSO	75%zero	sd=1	0.848 (0.056)	7.784 (10.186)	12.25 (1.209)
Hi-LASSO	75%zero	sd=3	0.812 (0.052)	8.769 (12.15)	11.1 (1.889)
Hi-LASSO	75%zero	sd=5	0.783 (0.069)	12.605 (23.071)	9.3 (1.559)
Hi-LASSO	overall	sd=1	0.713 (0.111)	22.772 (80.761)	10.883 (2.366)
Hi-LASSO	overall	sd=3	0.696 (0.098)	11.448 (19.523)	10.167 (2.437)
Hi-LASSO	overall	sd=5	0.691 (0.088)	8.132 (14.662)	9.9 (2.105)
Elastic net	25%zero	sd=1	0.567 (0.062)	1.502 (1.441)	52.4 (2.798)
Elastic net	25%zero	sd=3	0.537 (0.066)	1.48 (1.614)	53.15 (3.588)
Elastic net	25%zero	sd=5	0.51 (0.042)	1.245 (0.852)	54.3 (3.895)
Elastic net	50%zero	sd=1	0.598 (0.08)	1.84 (1.33)	44.6 (5.807)
Elastic net	50%zero	sd=3	0.517 (0.068)	1.755 (1.461)	49.1 (5.067)
Elastic net	50%zero	sd=5	0.528 (0.066)	1.972 (1.5)	45.8 (7.12)
Elastic net	75%zero	sd=1	0.543 (0.065)	2.317 (2.346)	42.9 (7.419)
Elastic net	75%zero	sd=3	0.547 (0.086)	3.723 (3.661)	37.35 (6.667)
Elastic net	75%zero	sd=5	0.578 (0.071)	4.671 (4.087)	31.55 (9.191)
Elastic net	overall	sd=1	0.569 (0.072)	1.886 (1.768)	46.633 (6.965)
Elastic net	overall	sd=3	0.534 (0.074)	2.319 (2.618)	46.533 (8.508)
Elastic net	overall	sd=5	0.539 (0.067)	2.629 (2.924)	43.883 (11.747)
Adaptive elastic net	25%zero	sd=1	0.84 (0.078)	1.551 (1.521)	41.6 (3.283)
Adaptive elastic net	25%zero	sd=3	0.729 (0.099)	1.323 (1.504)	43.65 (5.412)
Adaptive elastic net	25%zero	sd=5	0.719 (0.101)	1.199 (0.787)	42.35 (4.368)
Adaptive elastic net	50%zero	sd=1	0.816 (0.065)	1.857 (1.408)	29.85 (5.204)
Adaptive elastic net	50%zero	sd=3	0.721 (0.082)	1.987 (2.78)	31.85 (6.15)

Adaptive elastic net	50%zero	sd=5	0.716 (0.066)	2.12 (1.648)	31.35 (6.854)
Adaptive elastic net	75%zero	sd=1	0.726 (0.094)	2.164 (2.364)	23.05 (7.864)
Adaptive elastic net	75%zero	sd=3	0.665 (0.108)	3.592 (3.745)	26.15 (7.903)
Adaptive elastic net	75%zero	sd=5	0.668 (0.091)	4.866 (4.814)	21.3 (9.9)
Adaptive elastic net	overall	sd=1	0.794 (0.093)	1.857 (1.802)	31.5 (9.582)
Adaptive elastic net	overall	sd=3	0.705 (0.099)	2.301 (2.942)	33.883 (9.784)
Adaptive elastic net	overall	sd=5	0.701 (0.089)	2.728 (3.317)	31.667 (11.313)
RENBOOT	25%zero	sd=1	0.705 (0.045)	NA (NA)	21.65 (3.884)
RENBOOT	25%zero	sd=3	0.722 (0.046)	NA (NA)	20.95 (3.605)
RENBOOT	25%zero	sd=5	0.704 (0.064)	NA (NA)	21.4 (3.844)
RENBOOT	50%zero	sd=1	0.731 (0.062)	NA (NA)	19.45 (4.559)
RENBOOT	50%zero	sd=3	0.721 (0.036)	NA (NA)	16.95 (3.98)
RENBOOT	50%zero	sd=5	0.731 (0.039)	NA (NA)	16.95 (3.72)
RENBOOT	75%zero	sd=1	0.714 (0.087)	NA (NA)	16.55 (6.245)
RENBOOT	75%zero	sd=3	0.708 (0.063)	NA (NA)	14.3 (6.57)
RENBOOT	75%zero	sd=5	0.744 (0.063)	NA (NA)	10.25 (4.494)
RENBOOT	overall	sd=1	0.717 (0.067)	NA (NA)	19.217 (5.343)
RENBOOT	overall	sd=3	0.717 (0.049)	NA (NA)	17.4 (5.549)
RENBOOT	overall	sd=5	0.726 (0.058)	NA (NA)	16.2 (6.089)
BERM	25%zero	sd=1	0.853 (0.121)	1.899 (1.208)	43.95 (4.662)
BERM	25%zero	sd=3	0.881 (0.044)	1.63 (1.493)	39.45 (2.585)
BERM	25%zero	sd=5	0.833 (0.087)	1.47 (0.85)	38.4 (4.235)
BERM	50%zero	sd=1	0.86 (0.066)	2.209 (1.983)	29.7 (4.001)

BERM	50%zero	sd=3	0.879 (0.044)	2.596 (1.743)	26.45 (2.762)
BERM	50%zero	sd=5	0.869 (0.05)	2.829 (2.276)	26.95 (2.837)
BERM	75%zero	sd=1	0.845 (0.068)	2.282 (2.42)	18.95 (3.873)
BERM	75%zero	sd=3	0.827 (0.078)	4.182 (4.212)	19.15 (2.661)
BERM	75%zero	sd=5	0.813 (0.056)	4.965 (5.54)	18 (3.228)
BERM	overall	sd=1	0.853 (0.087)	2.13 (1.911)	30.867 (11.118)
BERM	overall	sd=3	0.862 (0.062)	2.803 (2.922)	28.35 (8.864)
BERM	overall	sd=5	0.839 (0.069)	3.088 (3.727)	27.783 (9.089)

Supplementary Table 2. Performance comparison of BERM and existing methods in high-dimensional simulation scenarios under varying conditions of sparsity and noise. Results are evaluated across three criteria: feature selection accuracy, coefficient estimation, and the extent of under- and over-selection. The values before the parentheses represent the mean results across the 20 simulated datasets, while the values within parentheses indicate the standard deviations of these results.

**Method**	**Zero Proportion**	**Error Noise Level**	**Balanced Accuracy**	**MSE**	**Number of Selected Vars**
Lasso	25%zero	sd=1	0.534 (0.032)	6.901 (1.309)	247.7 (22.714)
Lasso	25%zero	sd=3	0.536 (0.023)	7.107 (1.535)	240.25 (17.317)
Lasso	25%zero	sd=5	0.531 (0.033)	7.492 (1.433)	239.9 (31.751)
Lasso	50%zero	sd=1	0.552 (0.031)	5.993 (1.092)	218.85 (22.383)
Lasso	50%zero	sd=3	0.549 (0.032)	6.108 (1.431)	213.9 (23.566)
Lasso	50%zero	sd=5	0.545 (0.024)	5.542 (1.4)	224.9 (23.987)
Lasso	75%zero	sd=1	0.583 (0.037)	5.164 (2.325)	162.2 (33.984)
Lasso	75%zero	sd=3	0.549 (0.037)	5.453 (1.823)	184.4 (33.183)
Lasso	75%zero	sd=5	0.542 (0.041)	6.717 (4.893)	178.95 (40.144)
Lasso	overall	sd=1	0.556 (0.039)	6.019 (1.786)	209.583 (44.521)
Lasso	overall	sd=3	0.545 (0.031)	6.223 (1.72)	212.85 (34.048)
Lasso	overall	sd=5	0.539 (0.033)	6.584 (3.107)	214.583 (41.384)
Adaptive lasso	25%zero	sd=1	0.558 (0.021)	8.64 (1.768)	175.75 (13.357)
Adaptive lasso	25%zero	sd=3	0.561 (0.02)	8.971 (2.456)	168.55 (22.123)
Adaptive lasso	25%zero	sd=5	0.552 (0.033)	8.953 (1.917)	170.3 (19.445)
Adaptive lasso	50%zero	sd=1	0.577 (0.024)	6.988 (1.342)	135.5 (15.736)
Adaptive lasso	50%zero	sd=3	0.57 (0.025)	7.645 (1.532)	127.6 (19.632)

Adaptive lasso	50%zero	sd=5	0.572 (0.021)	7.418 (2.933)	129.25 (25.745)
Adaptive lasso	75%zero	sd=1	0.597 (0.029)	7.388 (3.233)	79.9 (28.531)
Adaptive lasso	75%zero	sd=3	0.586 (0.027)	7.363 (2.801)	87.4 (25.232)
Adaptive lasso	75%zero	sd=5	0.583 (0.031)	8.676 (7.859)	82.35 (27.366)
Adaptive lasso	overall	sd=1	0.577 (0.029)	7.672 (2.336)	130.383 (44.382)
Adaptive lasso	overall	sd=3	0.572 (0.026)	7.993 (2.393)	127.85 (40.037)
Adaptive lasso	overall	sd=5	0.569 (0.031)	8.349 (4.93)	127.3 (43.467)
Random lasso	25%zero	sd=1	0.512 (0.015)	11.831 (1.16)	469.75 (10.208)
Random lasso	25%zero	sd=3	0.514 (0.014)	11.936 (1.166)	472.45 (5.844)
Random lasso	25%zero	sd=5	0.504 (0.016)	11.834 (1.033)	470.05 (8.236)
Random lasso	50%zero	sd=1	0.524 (0.015)	11.275 (1.258)	464.35 (7.066)
Random lasso	50%zero	sd=3	0.523 (0.019)	11.398 (1.085)	465.35 (7.021)
Random lasso	50%zero	sd=5	0.522 (0.011)	10.523 (1.205)	463 (18.122)
Random lasso	75%zero	sd=1	0.565 (0.013)	11.392 (3.395)	442.95 (7.529)
Random lasso	75%zero	sd=3	0.562 (0.016)	13.133 (6.962)	442.75 (11.22)
Random lasso	75%zero	sd=5	0.558 (0.014)	9.898 (2.103)	446.25 (9.899)
Random lasso	overall	sd=1	0.533 (0.027)	11.499 (2.171)	459.017 (14.286)
Random lasso	overall	sd=3	0.533 (0.026)	12.156 (4.118)	460.183 (15.182)
Random lasso	overall	sd=5	0.528 (0.027)	10.752 (1.702)	459.767 (16.14)
Hi-LASSO	25%zero	sd=1	0.549 (0.018)	9.666 (2.58)	95.25 (5.919)
Hi-LASSO	25%zero	sd=3	0.556 (0.016)	9.891 (3.037)	94.65 (5.274)
Hi-LASSO	25%zero	sd=5	0.549 (0.02)	10.276 (2.304)	94.15 (4.196)
Hi-LASSO	50%zero	sd=1	0.575 (0.018)	8.381 (1.892)	89.35 (5.402)

Hi-LASSO	50%zero	sd=3	0.571 (0.019)	8.036 (1.813)	90.55 (4.651)
Hi-LASSO	50%zero	sd=5	0.573 (0.02)	7.736 (2.026)	89.3 (5.079)
Hi-LASSO	75%zero	sd=1	0.614 (0.027)	6.652 (4.615)	82.55 (4.828)
Hi-LASSO	75%zero	sd=3	0.595 (0.021)	6.836 (2.481)	82.1 (6.078)
Hi-LASSO	75%zero	sd=5	0.604 (0.025)	7.387 (4.104)	79.5 (5.434)
Hi-LASSO	overall	sd=1	0.579 (0.034)	8.233 (3.421)	89.05 (7.455)
Hi-LASSO	overall	sd=3	0.574 (0.025)	8.254 (2.76)	89.1 (7.455)
Hi-LASSO	overall	sd=5	0.575 (0.031)	8.466 (3.185)	87.65 (7.826)
Elastic net	25%zero	sd=1	0.523 (0.03)	6.468 (1.041)	298.4 (33.341)
Elastic net	25%zero	sd=3	0.53 (0.029)	7.245 (1.523)	278.95 (40.416)
Elastic net	25%zero	sd=5	0.521 (0.029)	6.89 (1.09)	314.75 (28.89)
Elastic net	50%zero	sd=1	0.547 (0.034)	5.707 (0.971)	226 (25.518)
Elastic net	50%zero	sd=3	0.548 (0.033)	6.434 (1.583)	223.05 (33.373)
Elastic net	50%zero	sd=5	0.546 (0.025)	5.854 (1.648)	221.7 (33.932)
Elastic net	75%zero	sd=1	0.582 (0.039)	5.082 (2.288)	164.8 (36.246)
Elastic net	75%zero	sd=3	0.551 (0.033)	5.502 (1.911)	180.3 (34.4)
Elastic net	75%zero	sd=5	0.546 (0.041)	6.234 (2.573)	176.15 (37.78)
Elastic net	overall	sd=1	0.551 (0.042)	5.752 (1.633)	229.733 (63.428)
Elastic net	overall	sd=3	0.543 (0.033)	6.394 (1.802)	227.433 (54.083)
Elastic net	overall	sd=5	0.538 (0.034)	6.326 (1.891)	237.533 (66.952)
Adaptive elastic net	25%zero	sd=1	0.525 (0.038)	6.254 (1.09)	279.35 (33.336)
Adaptive elastic net	25%zero	sd=3	0.534 (0.03)	6.658 (1.186)	280.4 (30.562)
Adaptive elastic net	25%zero	sd=5	0.52 (0.03)	6.608 (1.081)	309.85 (30.826)

Adaptive elastic net	50%zero	sd=1	0.548 (0.033)	5.099 (0.814)	228.25 (26.318)
Adaptive elastic net	50%zero	sd=3	0.55 (0.033)	5.714 (1.459)	225.45 (35.307)
Adaptive elastic net	50%zero	sd=5	0.547 (0.02)	5.02 (1.293)	226.55 (32.058)
Adaptive elastic net	75%zero	sd=1	0.589 (0.035)	4.055 (1.891)	159.1 (34.22)
Adaptive elastic net	75%zero	sd=3	0.552 (0.034)	4.625 (1.601)	179.15 (31.401)
Adaptive elastic net	75%zero	sd=5	0.542 (0.044)	5.54 (2.423)	181.85 (33.159)
Adaptive elastic net	overall	sd=1	0.554 (0.044)	5.136 (1.602)	222.233 (58.543)
Adaptive elastic net	overall	sd=3	0.545 (0.033)	5.666 (1.633)	228.333 (52.55)
Adaptive elastic net	overall	sd=5	0.536 (0.034)	5.722 (1.802)	239.417 (62.066)
RENBOOT	25%zero	sd=1	0.53 (0.013)	NA (NA)	37.3 (8.998)
RENBOOT	25%zero	sd=3	0.529 (0.01)	NA (NA)	37.6 (7.789)
RENBOOT	25%zero	sd=5	0.529 (0.015)	NA (NA)	36.1 (14.899)
RENBOOT	50%zero	sd=1	0.534 (0.027)	NA (NA)	50.3 (16)
RENBOOT	50%zero	sd=3	0.537 (0.025)	NA (NA)	54.2 (18.836)
RENBOOT	50%zero	sd=5	0.542 (0.019)	NA (NA)	47.35 (12.82)
RENBOOT	75%zero	sd=1	0.559 (0.047)	NA (NA)	78.2 (45.169)
RENBOOT	75%zero	sd=3	0.568 (0.03)	NA (NA)	66.7 (38.418)
RENBOOT	75%zero	sd=5	0.571 (0.035)	NA (NA)	68.1 (42.356)
RENBOOT	overall	sd=1	0.541 (0.034)	NA (NA)	55.267 (32.583)
RENBOOT	overall	sd=3	0.544 (0.029)	NA (NA)	52.833 (27.451)
RENBOOT	overall	sd=5	0.547 (0.03)	NA (NA)	50.517 (29.678)
BERM	25%zero	sd=1	0.612 (0.032)	6.953 (0.968)	250.4 (16.109)
BERM	25%zero	sd=3	0.6 (0.022)	7.372 (1.234)	250.25 (16.134)

BERM	25%zero	sd=5	0.596 (0.037)	7.28 (1.083)	254.85 (19.494)
BERM	50%zero	sd=1	0.602 (0.026)	5.48 (1.21)	202.75 (16.742)
BERM	50%zero	sd=3	0.599 (0.032)	6.039 (1.486)	200.95 (21.598)
BERM	50%zero	sd=5	0.6 (0.038)	5.455 (1.183)	207.1 (16.961)
BERM	75%zero	sd=1	0.618 (0.03)	4.878 (2.104)	147.7 (25.102)
BERM	75%zero	sd=3	0.588 (0.036)	5.496 (2.058)	164.2 (22.909)
BERM	75%zero	sd=5	0.576 (0.039)	6.22 (3.738)	165.5 (30.165)
BERM	overall	sd=1	0.611 (0.029)	5.77 (1.724)	200.283 (46.557)
BERM	overall	sd=3	0.596 (0.031)	6.303 (1.788)	205.133 (40.828)
BERM	overall	sd=5	0.591 (0.039)	6.318 (2.429)	209.15 (43.166)

Supplementary Table 3. computation times for various methods across datasets with moderate (60 variables and 300 observations) and high (500 variables and 300 observations) dimensions. We simulated 20 datasets for each scenario, incorporating varying noise levels (standard deviation, SD = 1, 3, 5) and zero proportions (25%, 50%, 75%). The values outside the parentheses indicate the mean computational time for all simulations, while the values inside the parentheses show the standard deviations of these times. All times are measured in minutes.

**Method**	**Moderate Dimensions**	**High Dimensions**
Lasso	0.007 (0.001)	0.012 (0.002)
Adaptive lasso	0.014 (0.001)	0.022 (0.002)
Random lasso	6.188 (0.31)	12.833 (2.722)
Hi-LASSO	3.382 (0.193)	7.615 (0.979)
Elastic net	0.099 (0.007)	0.181 (0.016)
Adaptive elastic net	0.19 (0.014)	1.841 (1.101)
RENBOOT	22.013 (1.92)	28.558 (2.007)
BERM	0.595 (0.041)	0.922 (0.056)
Unstructured BERM	7.731 (0.548)	12.392 (0.698)

Supplementary Table 4. Results of feature selection across BERM and unstructured BERM in both moderate and high-dimensional scenarios under extreme settings (e.g. 0% zero sparsity and 100% zero sparsity). Unstructured BERM refers to the tuning of α via cross-validation, as opposed to the fixed value of 0.5 used in BERM. We simulated 20 datasets for each scenario with varying noise levels (standard deviation, SD = 1, 3, 5). The values before the parentheses represent the mean results across the 20 simulated datasets, while the values within parentheses indicate the standard deviations of these results. In all non-zero scenarios, sensitivity is used to evaluate the feature selection; in all zero scenarios, specificity is applied to assess the feature selection.

**Dimensions**	**Method**	**Sparsity**	**Noise Level**	**Sensitivity/Specificity**
Moderate	Unstructured BERM	0%zero	sd=1	0.894 (0.037)
Moderate	Unstructured BERM	0%zero	sd=3	0.843 (0.038)
Moderate	Unstructured BERM	0%zero	sd=5	0.813 (0.055)
Moderate	BERM	0%zero	sd=1	0.907 (0.036)
Moderate	BERM	0%zero	sd=3	0.862 (0.052)
Moderate	BERM	0%zero	sd=5	0.833 (0.058)
Moderate	Unstructured BERM	100%zero	sd=1	0.889 (0.038)
Moderate	Unstructured BERM	100%zero	sd=3	0.892 (0.053)
Moderate	Unstructured BERM	100%zero	sd=5	0.892 (0.041)
Moderate	BERM	100%zero	sd=1	0.59 (0.152)
Moderate	BERM	100%zero	sd=3	0.56 (0.115)
Moderate	BERM	100%zero	sd=5	0.617 (0.203)
High	Unstructured BERM	0%zero	sd=1	0.464 (0.023)
High	Unstructured BERM	0%zero	sd=3	0.461 (0.03)
High	Unstructured BERM	0%zero	sd=5	0.458 (0.028)
High	BERM	0%zero	sd=1	0.539 (0.016)
High	BERM	0%zero	sd=3	0.536 (0.028)
High	BERM	0%zero	sd=5	0.541 (0.025)
High	Unstructured BERM	100%zero	sd=1	0.768 (0.038)
High	Unstructured BERM	100%zero	sd=3	0.757 (0.044)
High	Unstructured BERM	100%zero	sd=5	0.771 (0.044)
High	BERM	100%zero	sd=1	0.293 (0.061)
High	BERM	100%zero	sd=3	0.292 (0.023)
High	BERM	100%zero	sd=5	0.275 (0.028)
