# Supplementary material for: Bootstrap-enhanced regularization addressing multicollinearity and skewness in high-dimensional immunophenotyping data
Source: BMC Bioinformatics. 2026 Aug 6;27:259. doi: 10.1186/s12859-026-06586-x (PMC13729485; doi:10.1186/s12859-026-06586-x)
Supplement: Supplementary file 2 [file 12859_2026_6586_MOESM2_ESM.docx]

Supplementary Methods

**Details of implementations/settings for methods compared**

Lasso (Tibshirani, 1996) introduces an $L_{1}$-norm penalty to the linear regression objective function, which simultaneously selects variables and estimates their coefficients. Specifically, lasso solves the following optimization problem:

$$\min_{\beta} \sum_{i=1}^{n} \left( y_{i}-\sum_{j=1}^{p} \beta_{j}x_{\mathrm{ij}} \right)^{2}+\lambda\sum_{j=1}^{p} \left| \beta_{j} \right|$$

(2)

, where $\lambda$ is a non-negative regularization parameter that controls the strength of the penalty. When $\lambda$ is sufficiently large, some coefficients are shrunk to exactly zero, effectively performing variable selection. For implementation, we used the train() function from the caret R package (Kuhn, 2008). The glmnet() function from the glmnet R package (Friedman et al., 2010) was specified as the method, with $\alpha=1$ to enforce the lasso penalty. The optimal tuning parameter $\lambda$ was determined by exploring a sequence of values from ${10}^{2}$ to ${10}^{-3}$, decreasing by 0.1 on the log scale. The model with the smallest root mean square error (RMSE) based on 10-fold cross-validation was selected.

Expanding on the traditional Lasso, the adaptive lasso (Zou, 2006) modifies the regularization term by incorporating weights that adapt based on initial estimates from ordinary least squares (OLS) or ridge regression. The optimization problem is formulated as:

$$\min_{\beta} \sum_{i=1}^{n} \left( y_{i}-\sum_{j=1}^{p} \beta_{j}x_{\mathrm{ij}} \right)^{2}+\lambda\sum_{j=1}^{p} \hat{w}_{j}\left| \beta_{j} \right|$$

(3)

, where $\hat{w}_{j}=\frac{1}{{\hat{\beta_{j}}}^{\gamma}}$ , $\gamma>0.$The weights $\hat{w}_{j}$ inversely scale the penalty according to the magnitude of the initial estimates $\beta_{j}$. To address multicollinearity, we used ridge regression estimates as the initial weights. The train() function from the caret package (Kuhn, 2008), was employed to identify the optimal ridge regression parameters by setting α=0 and tuning parameter $\lambda$ over the same search space as above. The resulting ridge estimates were then used as weights $\hat{w}_{j}=\left| \frac{1}{\hat{\beta_{j}}} \right|$ in the lasso model, implemented by specifying the penalty.factor argument in the train() function.

Elastic net (Zou & Hastie, 2005) combines the $L_{1}$ and $L_{2}$ norm penalties of the lasso and ridge methods.

$$\min_{\beta} \frac{1}{2n}\sum_{i=1}^{n} \left( y_{i}-\sum_{j=1}^{p} \beta_{j}x_{\mathrm{ij}} \right)^{2}+\lambda\left( \frac{1-\alpha}{2}\sum_{j=1}^{p} \beta_{j}^{2}+\alpha\sum_{j=1}^{p} \left| \beta_{j} \right| \right)$$

(4)

, where $\lambda$ and $\alpha$ are non-negative tuning parameters. We used the train() function from the caret package (Kuhn, 2008) to determine the optimal $\lambda$ and $\alpha$. The search space for $\lambda$ ranged from ${10}^{2}$ to ${10}^{-3}$ in decrements of 0.1 on the log scale, while $\alpha$ was explored over 20 equally spaced values between 0 and 1. The optimal combination of $\lambda$ and $\alpha$ was selected based on the lowest RMSE observed during 10-fold cross-validation.

The adaptive elastic net (Zou & Zhang, 2009) extends the elastic net by incorporating adaptive weights, similar to the adaptive Lasso.

$$\min_{\beta} \frac{1}{2n}\sum_{i=1}^{n} \left( y_{i}-\sum_{j=1}^{p} \beta_{j}x_{\mathrm{ij}} \right)^{2}+\lambda\left( \frac{1-\alpha}{2}\sum_{j=1}^{p} \beta_{j}^{2}+\alpha\sum_{j=1}^{p} \hat{w}_{j}\left| \beta_{j} \right| \right)$$

(5)

, where $\hat{w}_{j}=\left| \frac{1}{\hat{\beta_{\mathrm{elnet}}}} \right|$  are derived from initial estimates obtained from a preliminary elastic net model. We used the caret package (Kuhn, 2008) for the initial estimation and the elnet_cv() function from the pense R package (Freue et al., 2019) to identify the optimal parameter combination. The process involved 10-fold cross-validation, with weights specified in the penalty_loadings argument.

Random lasso (Wang et al., 2011) is an extension of lasso for high-dimensional data using a two-stage implementation of bootstrapping and fitting lasso regressions on random subsets of predictors. In our implementation, we used 500 bootstrap resamples, randomly selecting 15%-20% of predictors in each iteration. The penalty parameter $\lambda$ was chosen using the same approach as in the standard Lasso method.

High-dimensional LASSO (Hi-LASSO) (Y. Kim et al., 2019) extends random lasso by refining coefficient estimation through bootstrapping and applying a statistical cutoff for feature selection. We implemented Hi-LASSO using the HiLasso() function from the hi_lasso Python package (Y. Kim et al., 2019) in Python 3.10.14. For moderate-dimensional datasets, 30 predictors were selected in each stage, while for high-dimensional datasets, this number was increased to 300. The number of bootstrap samples was set to 30 times the total number of predictors divided by the expected number of candidate variables, as recommended in the original manuscript (Y. Kim et al., 2019).

Robust Elastic Net via Bootstrap Method (RENBOOT) combines the elastic net with balanced bootstrap confidence intervals to identify important variables (H. Kim & Lee, 2021). Variables are considered irrelevant if their confidence intervals cover zero; otherwise, their importance is determined by the absolute averaged coefficients from bootstrap samples. We implemented RENBOOT using the balanced_bootstrap() function from the resamplr R package (Tim Hesterberg, 2014) with a resample size of 100. For each bootstrap sample, the optimal elastic net parameters $\lambda$ and $\alpha$ were determined via 10-fold cross-validation using the train() function. A 95% confidence interval for each coefficient was calculated using the bias-corrected and accelerated method (DiCiccio & Efron, 1996).

**Details of analysis of immunophenotyping data**

For the training of the model using the controls, we first imputed missing values for each feature using its median, followed by z-transform scaling. We set aside 10% of the data as the testing dataset. From the remaining data, 80% was used as the training dataset and 20% was used as the validation dataset. We implemented BERM with 500 bootstrap samples across 1,000 random train-test subset splits. The final variable coefficients were determined using the median of the estimated coefficients from BERM across all data splits.
